# Effects of small-molecule amyloid modulators on a *Drosophila* model of Parkinson’s disease

**DOI:** 10.1371/journal.pone.0184117

**Published:** 2017-09-01

**Authors:** Małgorzata Pokrzywa, Katarzyna Pawełek, Weronika Elżbieta Kucia, Szymon Sarbak, Erik Chorell, Fredrik Almqvist, Pernilla Wittung-Stafshede

**Affiliations:** 1 Airoptic Sp. z o.o., Poznań, Poland; 2 Department of Chemistry, Umeå University, Umeå, Sweden; 3 Department of Biology and Biological Engineering, Chalmers University of Technology, Gothenburg, Sweden; Stanford University School of Medicine, UNITED STATES

## Abstract

Alpha-synuclein (aS) amyloid formation is involved in Parkinson’s disease (PD); therefore, small molecules that target aS and affect its aggregation are of interest as future drug candidates. We recently reported modified ring-fused 2-pyridones that modulate aS amyloid formation *in vitro*. Here, we describe the effects of such molecules on behavioral parameters of a *Drosophila* model of PD (i.e., flies expressing human aS), using a new approach (implemented in a commercially available FlyTracker system) to quantify fly mobility. FlyTracker allows for automated analysis of walking and climbing locomotor behavior, as it collects large sequences of data over time in an unbiased manner. We found that the molecules *per se* have no toxic or kinetic effects on normal flies. Feeding aS-expressing flies with the amyloid-promoting molecule FN075, remarkably, resulted in increased fly mobility at early time points; however, this effect switched to reduced mobility at later time points, and flies had shorter life spans than controls. In contrast, an amyloid inhibitor increased both fly kinetics and life span. In agreement with increased aS amyloid formation, the FN075-fed flies had less soluble aS, and *in vitro* aS-FN075 interactions stimulated aS amyloid formation. In addition to a new quantitative approach to probe mobility (available in FlyTracker), our results imply that aS regulates brain activity such that initial removal (here, by FN075-triggered assembly of aS) allows for increased fly mobility.

## Introduction

Parkinson’s disease (PD) is the second most common neurological disorder and the most common movement disorder. It is characterized by widespread degeneration of subcortical structures of the brain, especially dopaminergic neurons in the substantia nigra. These changes are coupled with bradykinesia (slowness in execution and decrease of amplitude and range of movements), rigidity and tremor, resulting in difficulties in walking and abnormal gait in patients [1]. The assembly process of the intrinsically-unstructured protein α-synuclein (aS) has been linked to the molecular basis of PD. aS is a major component of the amyloid aggregates found in Lewy body inclusions, which are pathological hallmarks of PD, and duplications, triplications and point-mutations in the aS gene are related to familial PD cases [2, 3]. The exact function of aS is unknown, but aS is suggested to be involved in synaptic vesicle release and trafficking, physiological regulation of enzymes and transporters, and in cell survival by controlling the neuronal apoptotic response [4, 5]. aS appears to be present in soluble and membrane-associated forms at presynaptic nerve terminals [6–8].

aS can assemble via oligomeric intermediates to amyloid fibrils and, finally, to inclusion bodies (Lewy bodies) under pathological conditions [9]. Although soluble aS oligomers have been proposed to be the most toxic species in PD-related neurodegeneration [10, 11], recent work with pre-formed aS in fibrils has demonstrated that the amyloid fibrils themselves may be toxic and can amplify *in vivo*, transmit to other cells, and cross the blood-brain barrier [12–14]. Despite the lack of a mechanistic understanding of PD, many studies have focused on small synthetic or natural molecules that inhibit aS monomers to assemble into toxic oligomers and/or amyloid fibrils, or that divert the aS assembly process toward non-toxic aggregates, as an approach to counteract the disease [15, 16]. Inversely, the identification of small molecules that promote the aggregation of aS into oligomers and amyloid fibers could be helpful as research tools for the elucidation of early events during PD development in animal models. Most current animal models of PD are limited to studies of later events during disease progression, as they involve the use of toxic chemicals with non-aS targets that directly kill neurons [17–20].

FN075 is a low molecular weight peptidomimetic molecule that promotes aS amyloid formation *in vitro* via rapid formation of soluble oligomers [21]. Small-angle X-ray scattering (SAXS) data demonstrated that the FN075-initiated oligomers were structurally very similar to aS oligomers formed without FN075 and, as an indication of toxicity, they readily caused leakage of lipid vesicles *in vitro* [21, 22]. FN075 has a dihydro thiazolo ring-fused 2-pyridone central fragment designed to mimic a small C-terminal peptide with an extended β-sheet conformation [21, 23, 24]. A small chemical modification to the FN075 central fragment can change the properties so that the molecule becomes an inhibitor of aS aggregation [25]. We performed several *in vitro* characterizations of the designed 2-pyridone compounds on different amyloidogenic proteins, with an emphasis on aS [21, 24–26]. We recently extended our work to mice and injected FN075 into the brains of normal mice [27]. We discovered that a single dose of FN075, months later, promotes neuronal damage and symptoms similar to early PD. None of these effects were found upon the injection of an aS amyloid inhibitor molecule, or when FN075 was injected into aS knock-out mice [27]. In comparison to mice, fly models are attractive as they have a short life cycle, very low comparative costs and allow for powerful genetic manipulations [28]. It has been shown that several fly models recapitulate the essential features of PD [28] upon aS (wild-type and disease mutants) over-expression [1, 29]. As evidenced previously, it causes selective and progressive loss of dopaminergic neurons that are associated with the presence of aS inclusions in the form of Lewy bodies and Lewy neurites. Global neural expression of aS is also reported to cause gradual behavioral and locomotion defects, without having an impact on fly longevity [28, 30].

In the present study, we used three molecules with the same ring-fused 2-pyridone central fragment: FN075 that promotes aS amyloid (described above), one inhibitor (MS400) that is structurally very similar to FN075 but carries an amine in position C6 [25], and a control 2-pyridone molecule (C10) that has no effect on aS amyloid *in vitro* (S1B Fig). The effects of these molecules together with the established drug, L-Dopa, have been studied when fed to *Drosophila* flies. For this, we applied a new technology to better quantify fly activity, named FlyTracker. This is an automated video tracking system that allows for simultaneous recording and measurement of locomotor behavior in modular array by capturing images over several fly tubes (S1A Fig). This system is unique in comparison to other developed fly tracking systems, where the number of tracked flies is limited to one vial [31–33]. We found that *Drosophila* flies over-expressing human aS and fed with FN075, notably, became more active at a younger age than the vehicle-treated flies. However, the effect was reversed with age, and the FN075-fed flies ended up with shorter life spans than the vehicle- and inhibitor-fed aS-expressing flies. *In vitro* experiments and aS quantification in the fly brains supported the finding that the FN075 effects were caused by aS interactions.

## Materials and methods

### *Drosophila* stocks

Expression of wild type aS (Bloomington Drosophila Stock Center BDSC, Indiana University, stock #8146; w*; *P{w*^*+mC*^ = *UAS-Hsap\SNCA*.*F}5B*) was achieved with a *nSyb-Gal4#2–1* driver line used previously [34], where expression of Gal4 transcriptional activator was under the regulatory control of the neuronal synaptobrevin (*nSyb*) gene located on the third chromosome (kind gift of Dr. Julie Simpson; Howard Hughes Medical Institute, MD). In control experiments, we used wild-type Oregon-R strain originally obtained from the BDSC #6361 and crossed with the *nSyb-GAL4* (*w*^+^; +; +/*nSyb-GAL4*, denoted CTRL VEH), as well as the following genotypes: *w; +; UAS-Hsap/+*, denoted CTRL TG VEH and aS-expressing flies *w; +; UAS-Hsap/nSyb-Gal4*, denoted AS VEH. Prior to experiments, the genetic backgrounds of all strains were equilibrated to that of the *w1118* by five generations of out-crossing.

### Fly rearing and drug feeding assay

Flies were kept at 60% humidity at +20°C under a 12:12 h light:dark cycle (8 a.m. to 8 p.m. daily) until eclosion and at +29°C post eclosion. This temperature shift was adopted to lower the expression of aS during development before adding the tested compounds. The crossings were reared in bottles containing standard *Drosophila* food (corn meal, corn syrup solids, yeast, water and agar). Newly eclosed female flies (10 flies per vial) were transferred into 5 ml ventilated vials (75 x 13 mm, polystyrene tubes with archiving caps with filter, Sarstedt, Nümbrecht, Germany), containing low-melt fly food and tested compounds according to the formula developed by [35] for mixing drugs in low volumes. Briefly, the food was prepared with distilled water containing 2% (wt/vol) autoclaved yeast, 7% (vol/vol) corn syrup liquids, and 1.5% (wt/vol) agarose (composed of 1 part standard agarose to 11 parts low-melt agarose). The food was mixed as a liquid with drugs at 37°C. The ring-fused 2-pyridone compounds (FN075, MS400, C10; chemical structures shown in S1B Fig) [21, 25] were dissolved in 95% ethanol and mixed into the low-melt fly food at appropriate concentrations (final 2.5% ethanol solvent). The resulting food-plus compound mixtures solidified at 30°C into soft fly-edible gels. L-Dopa (3,4-Dihydroxy-L-phenylalanine #D9628 Sigma-Aldrich, Saint Lois, MO) containing food at the final concentration of 1mM was made by mixing with ascorbic acid (25mg/100ml) and then adding into 37°C freshly made food. Ascorbic acid was used to prevent drug oxidation. For larval feeding regime, parental crosses were placed for 1 day in vials with standard *Drosophila* food, containing the respective drug at appropriate concentration (prepared as above). Larvae were allowed to feed and develop in the vials at 25°C; thereafter, the female progeny having both the *nSyb-Gal4* driver and *UAS-Hsap* (*w; +; UAS-Hsap/nSyb-Gal4*) were transferred to 5 ml ventilated vials (10 flies per vial) containing low-melt fly food and tested compounds, and continued as in adult fly feeding regime. Every 2–3 days, the flies were transferred to fresh vials, and the number of dead flies was recorded throughout the lifetime of all flies. Graphs and statistical comparisons were generated with IBM SPSS 20 Statistics (IBM Corporation, Armonk, NY).

### Locomotion tracking with FlyTracker

Fly locomotion was tracked using FlyTracker (a new system commercially available from Airoptic Sp. z o.o., Poland; product number FT10.01.04–AAA; URL http://www.airoptic.pl/en/products), as described below and in the Results section. Briefly, the FlyTracker apparatus consists of a plastic frame, which incorporates a VGA camera (640 x 480 pixels resolution) and a tube holder rack. The camera is fixed at a distance of 105 mm from the center of the tubes and is connected to the computer with a USB interface (S1A Fig). Images over 10 consecutive seconds from four individual vials were simultaneously acquired at 30 frames per second and quantified using the accompanying dedicated FlyTracker Windows-based software. This software consists of two modules: one for detection of individual flies and the other for tracking fly movements. Measurements of the fly movements (starting 10 female flies per vial) were recorded once per week until the time when all the flies had died or lost climbing ability. The number of surviving flies was counted each time. The locomotor parameters computed by the FlyTracker system are described in the Results section. Recordings from each vial were acquired in two independent sequences, one after another, and the results were averaged; each treatment was run in 3–10 independent vials. The fly tube holder rack was tapped down three times before each trial to activate the locomotion. The freshly made food, containing drugs was changed every 2–3 days at 10 a.m. The flies were allowed to feed and accommodate to the vials in the incubator, and the readouts were carried out at 2 p.m. the same day the food was changed in order to ensure constant conditions.

### Protein aggregation *in vitro*

Freshly thawed sample of recombinant human wild-type aS at 1 mg/ml in buffered solution (phosphate buffer saline pH 7.4, 100 mM NaCl, 2.5% v/v EtOH) was incubated at 37°C with slight agitation (100 r.p.m.), with and without the addition of 100 μM FN075.

### ATR-FTIR measurements

The Attenuated Total Reflectance Fourier Transform Infrared (ATR-FTIR) spectra at room temperature was measured on a Nicolet iS50 FTIR spectrophotometer equipped with one pass diamond crystal ATR module (Thermo Fisher Scientific, Waltham, MA). The spectrometer was purged with N_2_ to remove the contribution of atmospheric water vapor and CO_2_ from all spectra. Each spectrum was an average of 100 scans at room temperature with a resolution of 2 cm^-1^ in the spectral range of 4000–400 cm^−1^. At indicated time points (0h–144h), 5 μl protein samples were taken out of the incubator and dropped on top of the ATR crystal. Raw data corresponding to amide-I region (1700–1600 cm^-1^) were deconvoluted by using the Fourier self-deconvolution (FSD) method. The deconvoluted spectra in the amide-I region were subsequently subject to Gaussian curve-fitting procedure in order to quantify the secondary structure content in aS. The water component was subtracted from each of the sample spectra. Data analysis was performed with Omnic program (Thermo Fisher Scientific, MA) and OriginPro 2015 (OriginLab Corporation, MA), according to the manufacturers’ instructions.

### Statistical data analysis

Graphs and statistical comparisons were generated with IBM SPSS 20 Statistics (IBM Corporation, Armonk, NY). Survival data were analyzed with Kaplan-Meier method, and statistical comparisons were made with log-rank pairwise analysis. Statistical significance for locomotor effects and ELISA tests was determined by General Linear Model multivariate analysis of variance (Multivariate GLM, also known as MANOVA), followed by Fisher's post hoc. The mean difference was considered to be statistically significant at the 95% confidence level. Final figures were assembled with Adobe Photoshop and Illustrator CC 2015.5 (Adobe Systems, San Jose, CA).

### aS extraction and quantification

For immunoblotting and ELISA, protein extracts were prepared according to the protocol modified from [36]. For details, see S1 Supporting Materials and Methods.

## Results

### FlyTracker system

The FlyTracker system is an automated video tracking system that consists of hardware and software (S1A Fig). The hardware is a single digital web-camera, mounted on a plastic chamber at a fixed distance (105 mm) from a custom designed fly tube holder rack. The camera is connected to a computer via a USB interface. The FlyTracker Windows-based software provides two modes of operation: a fly detector and a fly tracking mode based on algorithms parameterizing fly trajectories from acquired images. The system allows for simultaneous recording and measurement of locomotor behavior in modular array by capturing images over several independent fly tubes (four vials in this study). Despite the fact that our approach is based on a two-dimensional monitoring system, acquisition of data at 30 frames per second facilitates individual fly trajectories to be tracked and a number of computed locomotor parameters to be discerned. These include several quantitative measures extracted such as mean velocity, maximum velocity, total walking duration, total walking distance (i.e., total trajectory length), mean trajectory length per fly, percentage of time of fly motion, mean trajectory length per episode, mean number of trajectories and some others. In addition, the paramount feature of the FlyTracker software is an ability to compute the number of flies present in the vials. Thus it is possible to calculate and normalize the fly parameters despite constant changes in the number of flies in a vial. This normalization scheme implemented in the FlyTracker allows for a fast and robust estimation of vital behavioral parameters, necessary for sensitive screening of drug candidates. Our approach engages an application of small volume tubes requiring as little as 0.5 ml fly food that may be supplemented with small molecule compounds. These tubes easily accommodate 10 flies each, and special ventilation caps ensure a constant air exchange. The reduced need for large amounts of often expensive molecules makes the FlyTracker system a suitable method for high-throughput behavioral screening in forward genetic, candidate drug or toxicological screens. Here, we tested the FlyTracker system on a well-described fly model of sporadic PD to demonstrate its power as a sensitive method for investigation of the *in vivo* effects of small molecules, previously reported to modulate aS aggregation *in vitro*.

### Age-dependent climbing deficits in PD flies

**Fig 1:**
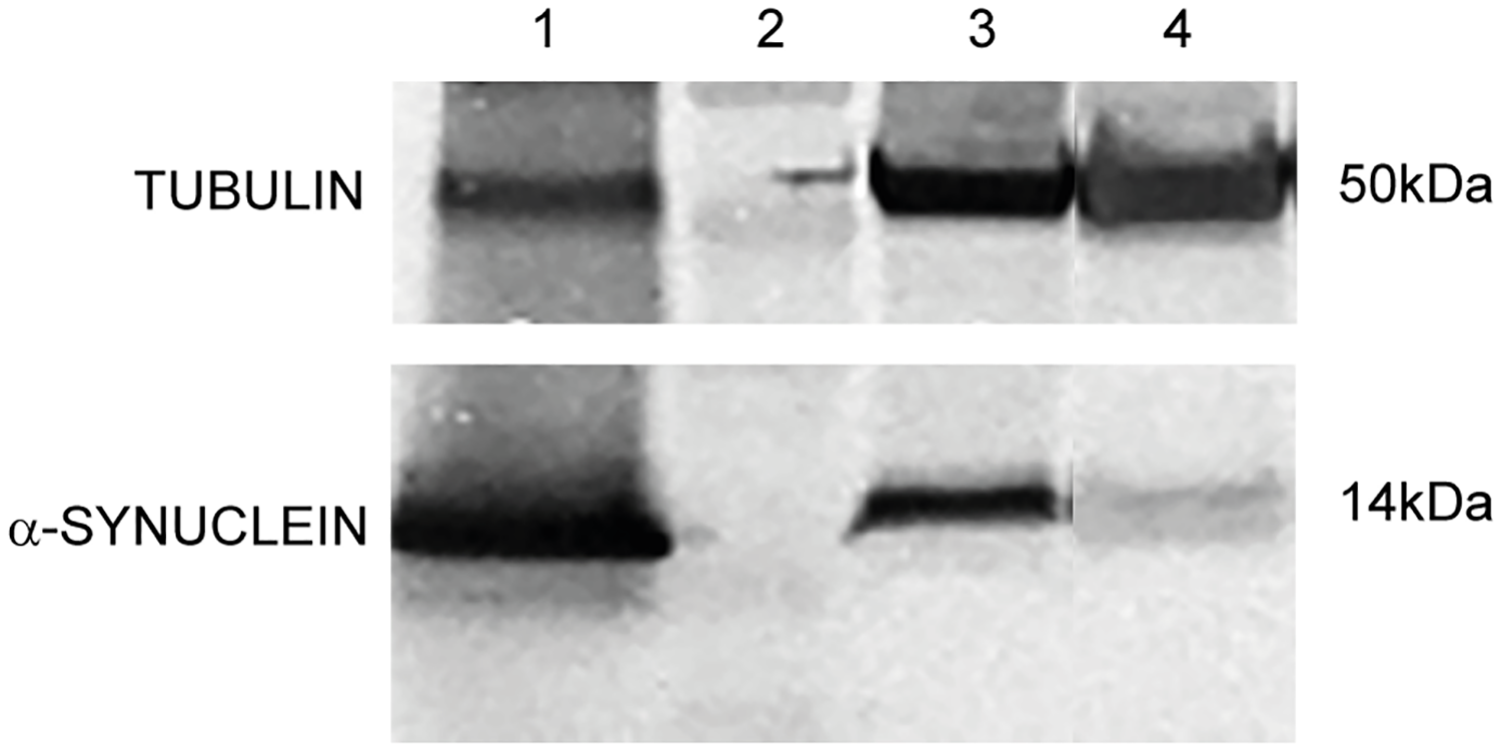
Western blot analysis of fly head protein extracts probed with antibody to human aS. The protein extracts are divided in soluble and insoluble aS fractions prepared as described in SI. Tubulin (upper panel), aS (lower panel). Lanes: 1. Recombinant human aS (5 ng), 2. Molecular weight marker, 3. Soluble fraction of fly head extracted aS, 4. Insoluble fraction of fly head extracted aS.

To ensure a high and robust expression of human aS, we used a neural synaptobrevin promoter (*nSyb-GAL4*), a type that was previously shown to yield about 60% increased aS levels compared to the standard and broadly used *elav-GAL4* neuronal promoter [1]. Protein head extracts probed with an antibody specific to human aS confirmed the presence of aS in both soluble and insoluble fractions (Fig 1). Signal intensity quantification allowed for estimation of aS expression levels, showing insoluble aS amount to be roughly 1/3 of that of soluble aS (S1C Fig; 3,4 ng soluble and 1,4 ng insoluble aS per fly head).

**Fig 2:**
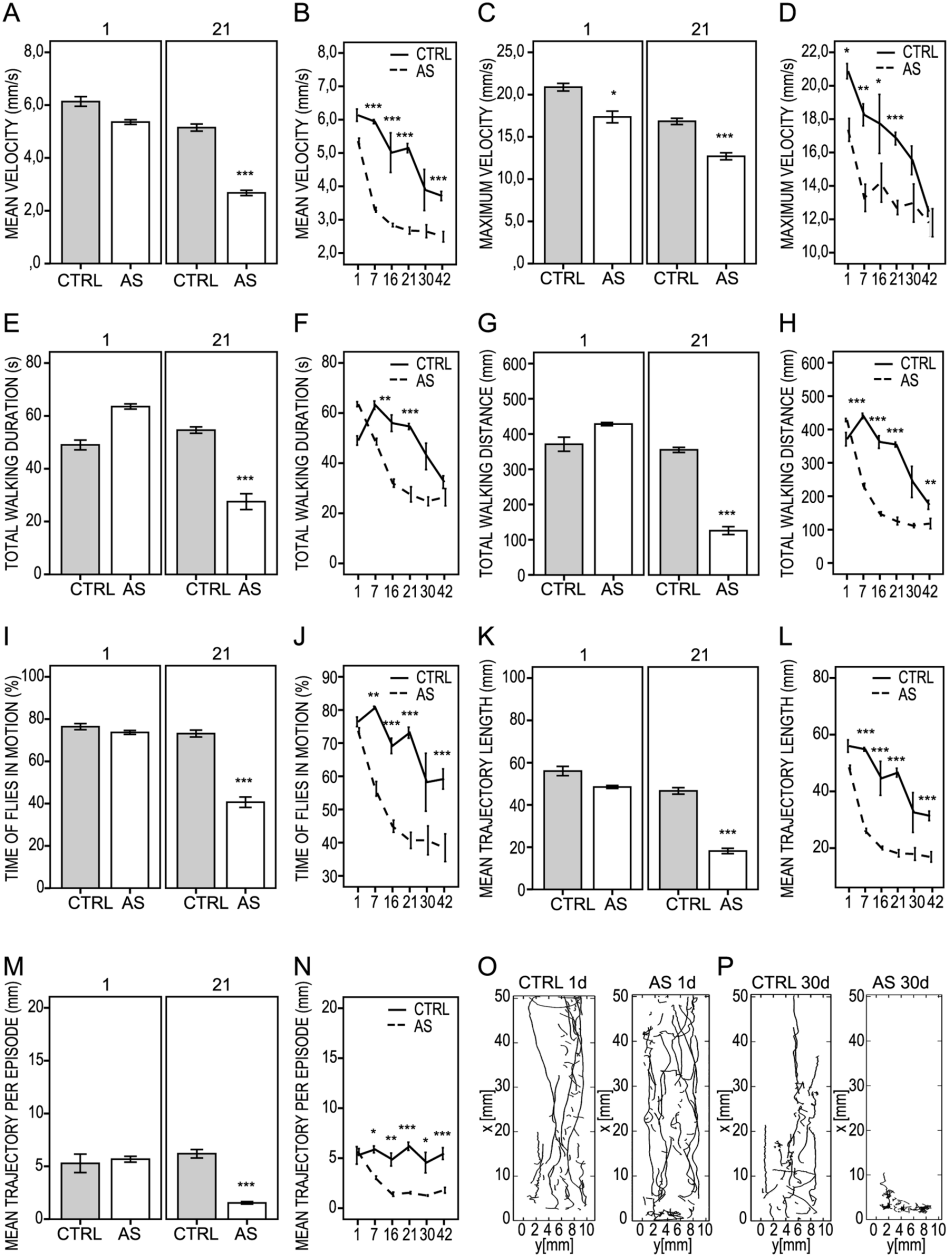
Kinetic parameters for control non-expressing *UAS-aS* flies (*w*; +; *UAS-Hsap*/+; CTRL) vs. aS-expressing flies (*w*; +; *UAS-Hsap/nSyb-Gal4*; AS) measured at 1, 7, 16, 21, 30 and 42 days of fly lifetime. (A, B) Mean velocity (mm/s). (C, D) Maximum velocity (mm/s). (E, F) Total walking duration (s). (G, H) Total walking distance (mm). (I, J) Percentage of time that flies are in motion (%). (K, L) Mean trajectory length (mm). (M, N) Mean trajectory length per episode (mm). (O, P) Sample tracings of fly trajectories for 1 day-young and 30-day old control and aS flies. Scattered line and bar diagrams represent the mean values. Error bars = ± SE. * *P* < 0.05; ** *P* < 0.01; *** *P* <0.001.

As evidenced previously, we also found that pan-neuronal expression of aS preserves the motion behavior in young flies but accelerates climbing deficits normally seen later in life in the control flies (Fig 2). This premature locomotor decline was earlier reported as being associated with intracellular accumulation of aS and the specific loss of dopaminergic neurons. Longevity, on the other hand, was shown to be insensitive to aS expression in flies [28, 30].

Quantification of fly trajectories with the FlyTracker system showed that subtle and non-significant changes between the aS-expressing and control flies were already present on day 1 and worsened with time as measured on days 7, 16, 21, 30 and 42 (Fig 2, for statistical comparison see S1 Table). This is reflected by the sharp drops in the mean and maximum velocity on day 7 in aS flies, and continuously decreasing each week until the flies became immobile around day 42 (Fig 2A–2D). The rate of decrease in the mean velocity was markedly different between the aS and the control flies, with drops from 5.6 mm/s down to 2.5 mm/s vs. 6mm/s to 5 mm/s for aS vs. control flies, respectively, within the first 3 weeks. Thereafter, the speed at which the aS flies moved leveled out, as values below 2 mm/s were not recognized as progressive fly movement (Fig 2A and 2B). Therefore, we set another parameter to quantify the percentage of time that the flies move faster than the cut-off speed of 2.5 mm/s. This value, described as the percentage of time that the flies are in motion (%), revealed no difference on day 1 but was significantly reduced on day 21 for the aS vs. the control flies (Fig 2I and 2J).

A significant reduction of other quantitative locomotion descriptors for aS flies was clearly apparent for the total walking distance and mean trajectory length (Fig 2G, 2H, 2K and 2L), as well as the mean trajectory length per episode (Fig 2M and 2N). Also, as expected, shorter trajectories and lower velocities were mirrored by a reduced duration of fly movement measured within the experimental time frame. Sample trajectories extracted by the FlyTracker software showing the effects of aS expression and aging on flies are presented in Fig 2O and 2P.

### Small molecule effects on fly life span

**Fig 3:**
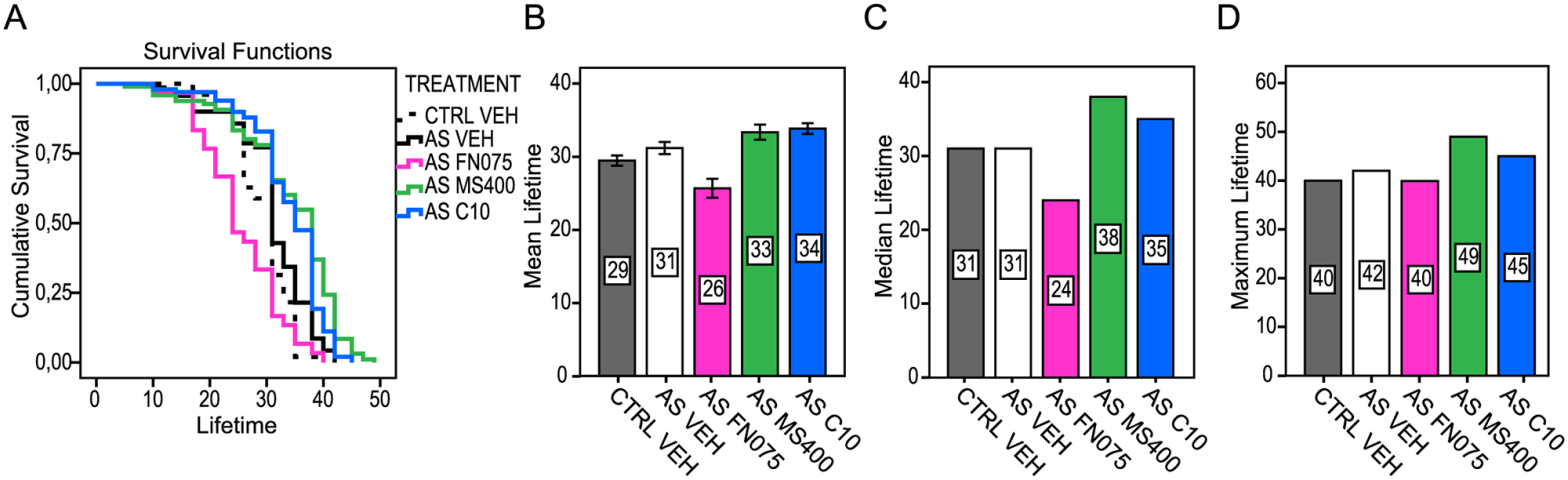
Feeding effects of 2-pyridones on aS-expressing flies life span. Survival analysis is presented by Kaplan-Meier curves. (A) Cumulative survival of control flies (*w*^+^; +; +/*nSyb-GAL4*; CTRL VEH, dotted black line) and aS expressing flies (*w*; +; *UAS-Hsap/nSyb-Gal4*; AS) treated with either vehicle (AS VEH, black) or compounds: FN075 (AS FN075, magenta), MS400 (AS MS400, green line) or C10 (AS C10, blue line) at 100 μM concentration. Bar diagrams show (B) mean, (C) median and (D) maximum lifetime. Numbers in bars represent days of mean, median and maximum lifetime (n = 10). Error bars imply ± SE.

As shown in S2A Fig, the selected molecules (FN075 and MS400, shown in S1B Fig) have no toxic effects on the development of aS-expressing and control flies. Similarly, these compounds did not affect the longevity of the control flies (S2B Fig, S2 Table). Nonsignificant changes were noted for the mean and median lifetimes that had no apparent effect on the maximum lifetime (S2C, S2D and S2E Fig). However, we found a reduced fly life span when feeding the adult aS flies with FN075, which was expected because FN075 gave mice PD-like symptoms [27], and it promoted aS amyloid formation *in vitro* [21]. In contrast, in accordance with being an aS amyloid inhibitor *in vitro* [25], the MS400 compound increased the mean, median as well as the maximum lifetime compared to the vehicle-treated aS and control flies (Fig 3). Both treatments induced significant changes in the life span (log rank analysis, supplementary S3 Table). In parallel, we used the molecule C10, with the same core fragment as FN075 and MS400 but differently substituted, as a negative control because it had no effect on aS amyloid formation *in vitro* (S1B Fig). This compound, like FN075 and MS400, had no toxic effects on the development of aS or control flies (S2A Fig), nor did it change the life span of the control flies (S2B, S2D and S2E Fig, S2 Table). Feeding adult aS flies with C10 slightly prolonged the maximum life span and increased the mean and median life spans (Fig 3, S3 Table).

### Small molecule kinetic effects on aS flies

**Fig 4:**
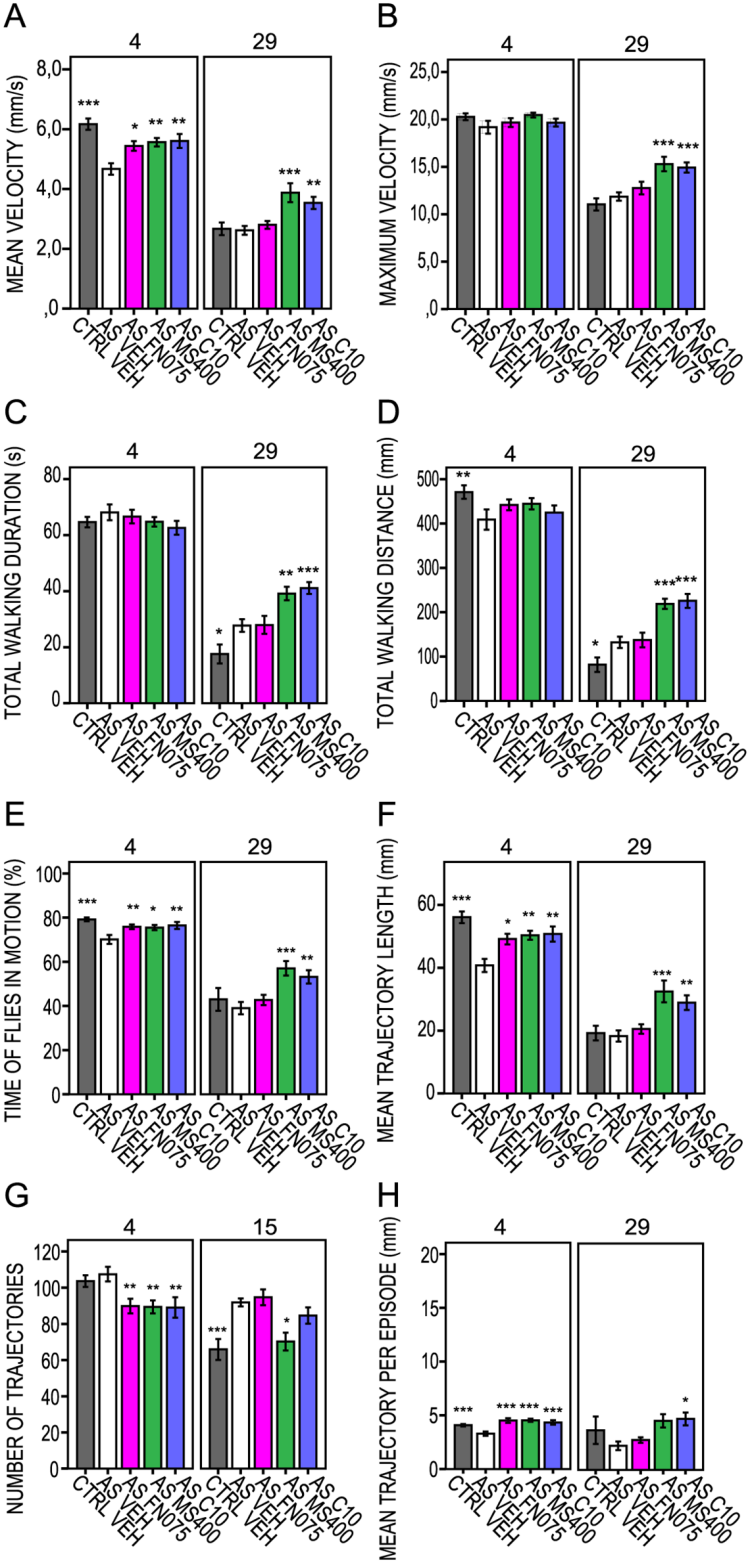
FlyTracker acquired kinetic parameters for aS flies measured at 4 and 29 days at adult drug feeding regime. Vehicle-treated aS-expressing flies (*w*; +; *UAS-Hsap/nSyb-Gal4*; AS VEH, white bars) and control flies (*w*^+^; +; +/*nSyb-GAL4*; CTRL VEH, gray bars). (A) Mean velocity (mm/s). (B) Maximum velocity (mm/s). (C) Total walking duration (s). (D) Total walking distance (mm). (E) Percentage of time that flies are in motion (%). (F) Mean trajectory length (mm). (G) Number of fly walking trajectories. (H) Mean trajectory length per episode (mm). Bars represent mean values (n = 10). Error bars indicate ± SE. * *P* < 0.05; ** *P* < 0.01; *** *P* <0.001. Tested compounds were either FN075 (AS FN075, magenta bars), MS400 (AS MS400, green bars) or C10 (AS C10, blue bars) at 100μM concentration.

When assessing the kinetic effects of molecules fed to adult flies (Fig 4, S3 Fig, S4 Table), we found that FN075 resulted in early effects with increases in mean velocity, mean trajectory length and increased percentage of time that the flies were in motion on day 4 and 8 (Fig 4C, 4E, and 4F, respectively). MS400, on the other hand, improved the mean and maximum velocities (Fig 4A and 4B), increased total walking distance (Fig 4C) and mean trajectory length (Fig 4F), increased the percentage of time that the flies were in motion (Fig 4E) and increased the mean trajectory length per episode up to day 29 (Fig 4H). Feeding with the control molecule C10 had initial effects on the locomotion parameters of the aS flies, but these faded out during the course of the trial. On day 29, however, most of the C10-related kinetic descriptors were elevated in comparison with the vehicle-treated aS flies. We note that the detected C10 effects on flies are in conflict with the lack of C10 modulation of aS amyloid formation *in vitro*. It is thus possible there are non-amyloid-related C10 effects on aS in the flies, which need further investigation to be explained.

**Fig 5:**
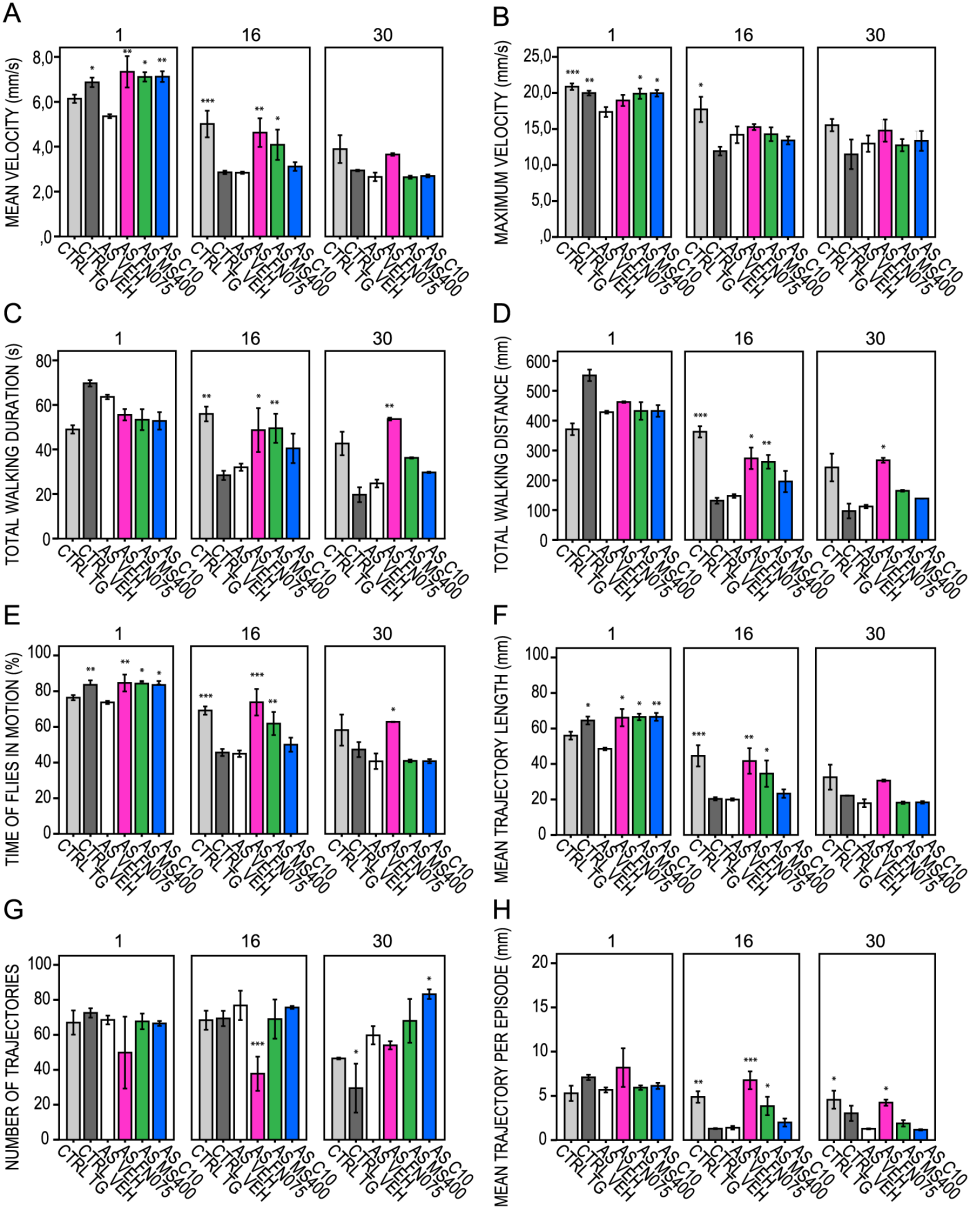
FlyTracker acquired kinetic parameters for controls and aS-expressing flies measured at 1, 16, 30 days of larval drug feeding regime. Controls: *nSyb-Gal4#2–1* outcrossed with Oregon R (*w*^+^; +; +/*nSyb-GAL4*; CTRL VEH, dark gray bars); non-expressing *UAS–aS* (*w*; +; *UAS-Hsap*/+; CTRL TG, light gray bars) and aS-expressing vehicle-treated flies (*w*; +; *UAS-Hsap/nSyb-Gal4*; AS VEH, white bars). (A) Mean velocity (mm/s). (B) Maximum velocity (mm/s). (C) Total walking duration (s). (D) Total walking distance (mm). (E) Percentage of time that flies are in motion (%). (F) Mean trajectory length (mm). (G) Number of fly walking trajectories. (H) Mean trajectory length per episode (mm). Error bars indicate ± SE. * *P* < 0.05; ** *P* < 0.01; *** *P* <0.001. Tested compounds were either FN075 (AS FN075, magenta bars) or MS400 (AS MS400, green bars) or C10 (AS C10, blue bars) at 100μM concentration.

In contrast, when the aS flies were treated with molecules already at the larval feeding stage, the kinetic parameters significantly improved for both FN075 and MS400, at least up to day 21 (Fig 5, S4 Fig, S5 Table). This elevated effect was sustained for FN075 until day 30, and brought all kinetic parameters to the level of the control non-aS expressing flies (CTRL TG). C10 showed no evident effects on aS fly locomotive behavior when fed already at the larvae stage. To assure that feeding with molecules did not result in general (non-aS mediated) effects on fly locomotion, we ran control experiments with aS non-expressing flies. Quantification of the extracted fly trajectories revealed that they were similar and independent of the precise chemistry of the small molecule fed to the flies. Most of the kinetic parameters showed no significant changes compared to the vehicle-treated control (S5 Fig, S6 Table). Thus, we conclude that the specific and divergent effects found for the molecules require aS expression.

### *In vitro* complementary analyses of FN075

**Fig 6:**
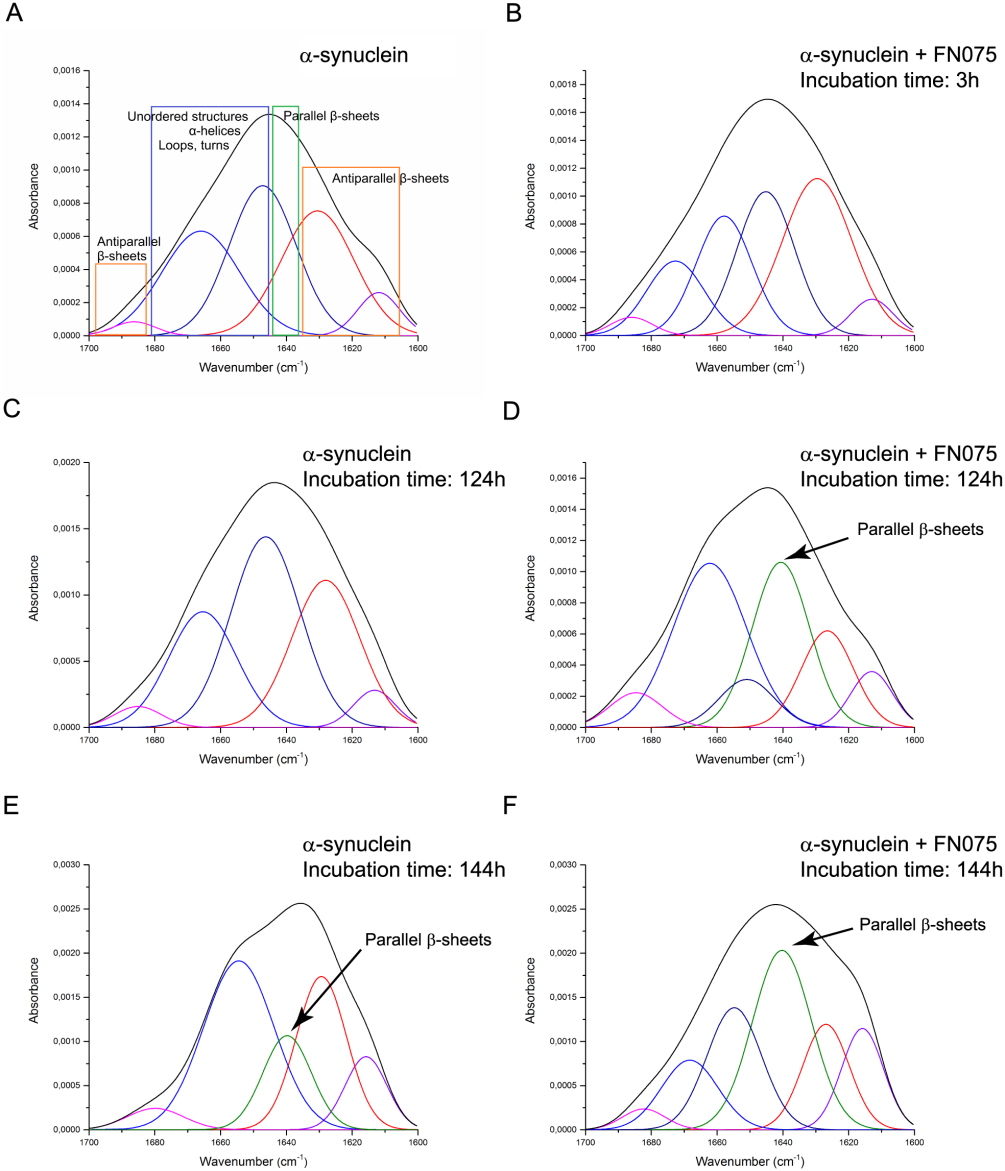
ATR-FTIR spectra of amide-I region of recombinant human aS. Secondary structure analyses were performed for aS incubated at 37°C alone (A, C, E) or with FN075 (B, D, F). Spectra were collected at different points of incubation: 0h, 3h, 124h and 144h. Deconvolution and curve-fitting were used to determine the secondary structure composition.

To confirm the previously reported interaction between the aS and FN075 *in vitro* [21, 25], we employed ATR-FTIR to probe conformational changes of purified aS (Fig 6). ATR-FTIR is a sensitive method for measurements of protein secondary structure and can be used to study protein aggregation *in vitro* [37]. The kinetics of aS amyloid formation is reflected by changes in the secondary structure content where oligomers encompass anti-parallel β-sheet structures and amyloid fibrils are characterized by parallel β-sheet content. FTIR analysis of aS incubated at 37°C with agitation was performed in the amide-I band, i.e., in the range of 1600 cm^-1^ to 1700 cm^-1^ as a function of time. Deconvolution and curve-fitting of the spectra for aS alone and aS in the presence of FN075 are shown in Fig 6A–6F. After immediate sample preparation (time 0 h), soluble aS (Fig 6A) showed distinguished bands in the amide-I region, which correspond mostly to disordered structures, loops, turns and α-helices (1673–1646 cm^-1^), random coil-like structures (major absorption at 1647 cm^-1^), and to β-sheet structures (1641–1612 cm^-1^). After 124 h of incubation, the intensity at 1647 cm^-1^ further increased at a disadvantage of other structures (Fig 5C). Finally, at 144 h, an absorption peak at 1640 cm^-1^, typical of parallel β-sheets, and thus amyloid fibrils, appeared (Fig 6E). For aS incubated with FN075, the absorption peak characteristic of amyloid fibrils appeared already at 124 h (Fig 6D) and was further increased at 144 h (Fig 6F). Although aS aggregation is slower here than in previous reports [21, 25, 37], which we explain by the fact that less agitation was applied and the lack of glass beads, importantly, the accelerating effect of FN075 remains.

**Fig 7:**
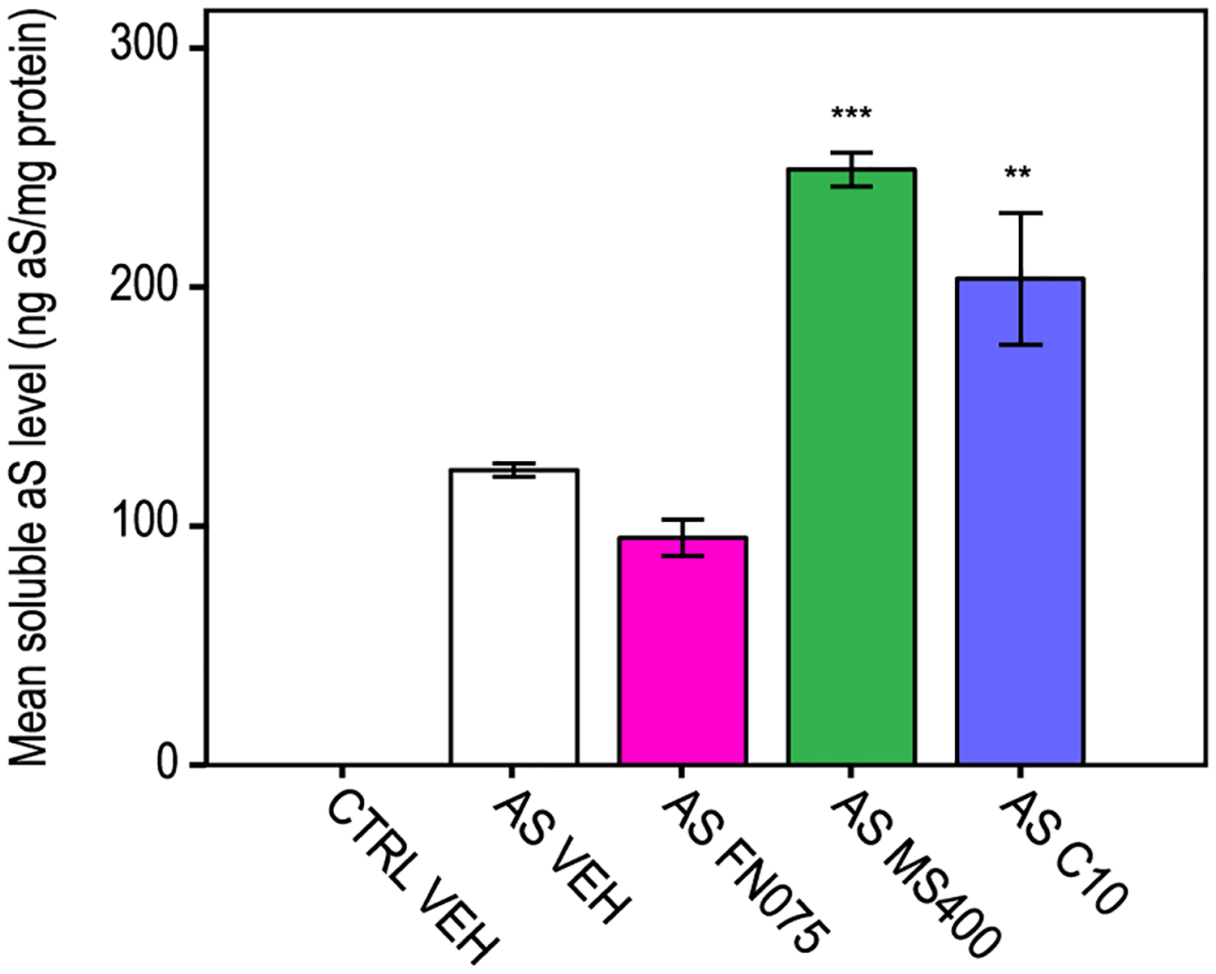
Effects of 2-pyridones on level of soluble aS in fly brain extracts of 20 days old flies. Mean soluble aS levels were measured with antibody specific to human aS in ELISA test. Controls: vehicle-treated nsyb-Gal4 outcrossed with Oregon R flies (CTRL VEH) or aS expressing flies (AS VEH, white bars). Tested compounds were FN075 (AS FN075, magenta bars) or MS400 (AS MS400, green bars) or C10 (AS C10, blue bars) at 100μM concentration. Bars represent mean values (n = 2). Error bars indicate ± SD. * *P* < 0.05; ** *P* < 0.01; *** *P* <0.001. Multivariate GLM followed by Fisher's post hoc showed *P* = 0.084 for AS FN075 vs AS VEH, *P*<0.001 for AS MS400 vs AS VEH and *P* = 0.002 for AS C10 vs AS VEH. For raw data see S7 Table.

The fact that FN075 interacts with aS in the flies such that aS aggregation results is indicated from analysis of the soluble amount of aS in fly brain extracts by ELISA. As expected, if FN075 promotes aS aggregation and insolubility, we find less soluble aS in the FN075 fed flies in contrast to the MS400 treatment (Fig 7; for statistical analysis see S7 Table). These data are in agreement with our previous work, which complemented the mice studies, where we analyzed aS brain content of FN075 and MS400 fed flies by Western blot [27]. Moreover, in aS knock-out mice, there were no effects of FN075 treatment on brain cell death, motor skills and serum metabolite pattern at 6 months after the injection.

### Testing a known compound, dopamine precursor

**Fig 8:**
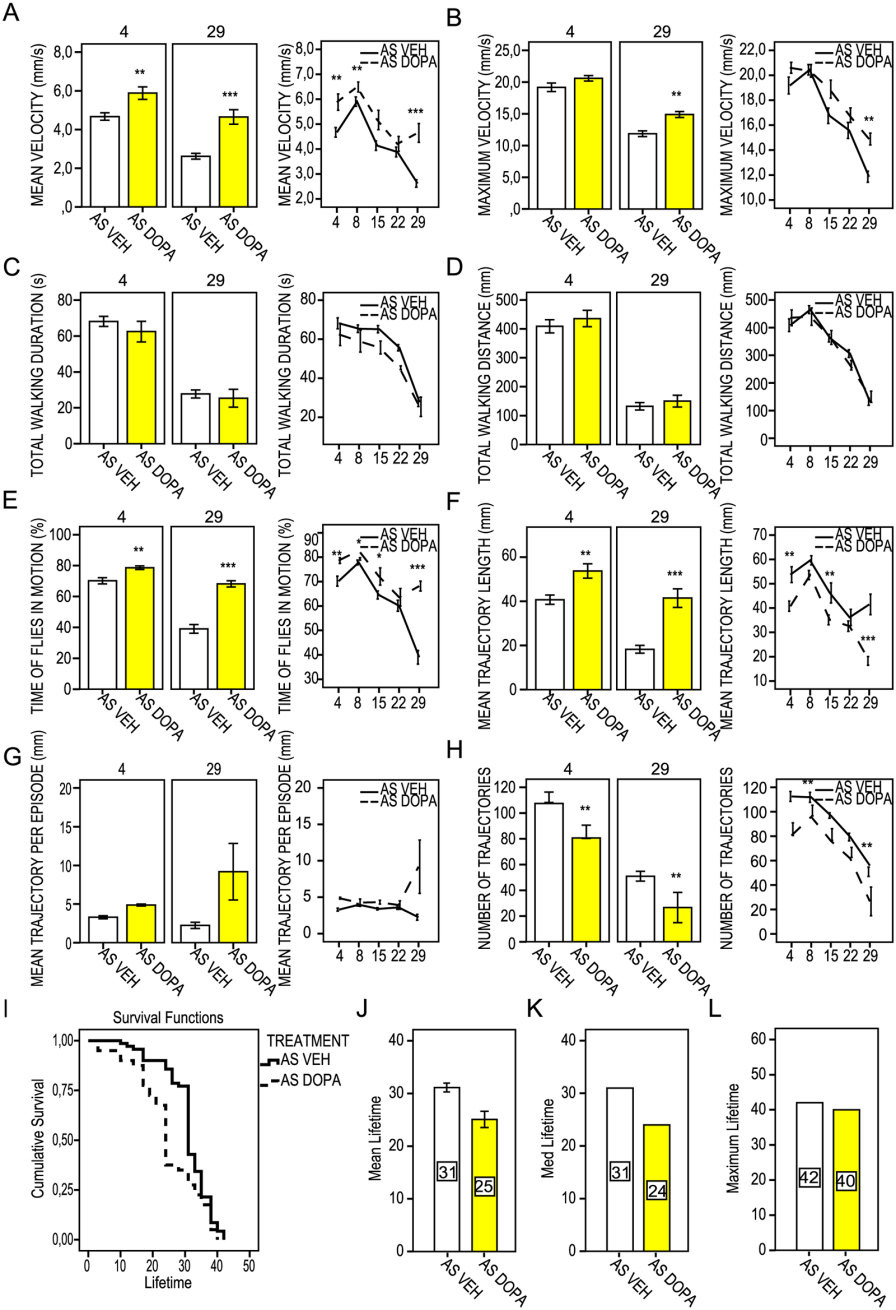
FlyTracker acquired kinetic parameters for control aS-expressing flies measured at 4, 8, 15, 22 and 29 days of adult fly drug feeding regime treated with either vehicle (*w*; +; *UAS-Hsap/nSyb-Gal4*; AS VEH, white bars) or with L-DOPA (*w*; +; *UAS-Hsap/nSyb-Gal4*; AS DOPA, yellow bars) at the final concentration of 1mM. Scattered line and bar diagrams represent mean values (n = 10). Error bars indicate ± SE. * *P* < 0.05; ** *P* < 0.01; *** *P* <0.001. (A) Mean velocity (mm/s). (B) Maximum velocity (mm/s). (C) Total walking duration (s). (D) Total trajectory length (mm). (E) Fly motion (%). (F) Mean trajectory length (mm). (G) Mean trajectory length per episode (mm). (H) Number of fly walking trajectories. (I) Cumulative survival of vehicle-treated aS flies (*w*; +; *UAS-Hsap/nSyb-Gal4*; AS VEH) and aS flies treated with L-DOPA (*w*; +; *UAS-Hsap/nSyb-Gal4*; AS DOPA). (J) Mean, (K) median and (L) maximum lifetime. Numbers in bars represent days of mean, median and maximum lifetime.

L-Dopa is a dopamine precursor and a common therapeutic used for PD [38, 39]. In our study, aS-expressing flies that fed on a diet of L-Dopa (Fig 8 and S8 Table) showed an increased mean velocity along the course of the trial (Fig 8A). This treatment improved the fly trajectories, seen as extended mean length of trajectories and decreased number of trajectories (Fig 8F and 8H), which was inversely correlated. Although L-Dopa improved the percentage of time that the flies were in motion already on day 4 and further corrected the fly motion index by a factor of two on day 29 (Fig 8E), it shortened the mean, median and maximum life span of the aS flies (Fig 8I–8L). Interestingly, there was no significant difference in the life span between L-Dopa and FN075-treated flies (for log-rank pair-wise comparisons see S9 Table).

## Discussion

Animal models of human diseases are helpful tools to not only evaluate the potential drug concepts but also to study the underlying mechanisms of human diseases. PD is a motor neurodegenerative disease that is becoming more and more common in the population as we become older. Although we have clues as to what proteins and reactions go awry and what the clinical manifestations are, many fundamental aspects of PD (such as what is the trigger, order of deleterious events, involved biochemical pathways, normal function of aS) remain elusive, and so far there is no cure for this disease. Therefore, new PD model and analysis systems allowing for complementary studies of biological consequences and putative drug intervention are of high importance.

Here, we presented a new way to probe motor skills of *Drosophila* flies and used this technology to assess the effects of small-molecule modulators (ring-fused 2-pyridones) of aS amyloid formation *in vitro*. The same molecules, recently tested in mice and amyloid-promoting FN075, were found to cause signs of PD at 6 months after injection, including altered serum metabolites, motor dysfunction in sticker-on-nose test and death of dopaminergic neurons [27]. In contrast, in the fly model, new aspects of drug treatment were evaluated. Here, motor function was probed differently, and the flies were monitored throughout their life span. We note that loss of dopaminergic cells in the aS-expressing flies as a function of small molecules was not tested here as previous reports have been contradictory and it is unclear if PD fly models involve the loss of dopamine-producing cells [28, 30, 40–43] or not [44–47]. The main discrepancies were found due misinterpretation of the decrease in TH immunostaining, or GFP signal, as a sign of neuronal loss without direct experimental evidence of cell death or occurrence of apoptotic processes in the PD fly models [44]. To avoid this uncertainty, we here focused specifically on defects in motor behavior, which is a key feature of PD. However, we note that future studies that reliably count TH-immunoreactive neurons as well as probe Lewy body pathology in the fly brains as a function of small molecule treatments are desired.

In aS-expressing flies fed with FN075, we found an increase in the motor activity at a young age (not captured in mice), followed by a reduction of activity upon aging and a shorter life span, as compared to control and inhibitor treated flies. The fact that FN075 acted on aS in the flies was supported by *in vitro* FTIR data, by the reduction of soluble levels of aS in brain extracts of FN075-treated flies, and by the fact that the molecules had no effects on flies lacking human aS. In analogy, we found that FN075 had no effect on the aS knock-out mice [27]. Our finding that the initial removal of aS (via FN075-triggered aggregation) has a *favorable* effect on fly neuronal activity is novel and suggests that aS moderates the neuronal activity under normal conditions [5].

Another surprising observation we made was that larvae feeding of FN075 resulted in solely positive effects on the flies. This may be explained by the induction of fly adaptive mechanisms (e.g., down-regulation of target genes) upon prolonged exposure to molecules [48], but our control experiments do not support this. Stage-specific differences between the adult- and larvae-fed flies have been noted for other compounds [49] and emphasize the importance of being aware of the fact that the experimental design may influence the outcome.

Fly models have been used to test the biological effects of many small molecules that modulate the aggregation of amyloidogenic proteins *in vitro*. For example, it was found that epicatechin gallate, a flavonoid antioxidant, delayed the loss of climbing ability and reduced oxidative stress in PD flies [50]. This, together with *in vitro* data of aS amyloid inhibition by this flavonoid, suggests that this natural product may have a beneficial effect on PD patients. Interestingly, in another study using a *Drosophila* model of Alzheimer’s disease (AD; i.e., over-expressing the amyloid-β peptide, Aβ) it was found that curcumin reduced neurotoxicity (reflected by increased locomotor activity and increased life span) by promoting Aβ fibrillation such that oligomers and pre-fibrillar species were diminished [51]. Treatment with L-Dopa, the dopamine precursor and the major drug used to mitigate the effects of dopaminergic neuron loss in PD patients, was previously shown to restore locomotor deficits in aS-expressing [1, 52] and dopamine-deficient flies [53, 54]. With respect to protein aggregation, dopamine can protect against aS amyloid formation *in vitro* [55]. However, if present in the cytoplasm, it can also make adducts with aS, and those can promote the accumulation of toxic cytoplasmic aS proto-fibrils, which would make the dopamine neurons being selectively vulnerable [55, 56]. Such a scenario may explain why we found that flies fed with L-Dopa do better, in terms of kinetics but still have a shorter life span than untreated aS flies. Notably, the L-Dopa results on the aS flies parallel the FN075 results as well as long-term treatment of PD patients with L-Dopa. In the latter case, patients initially show strong increases in their mobility, but after long-term treatment the patients often develop L-Dopa induced dyskinesia [57]. Many future studies are necessary to reveal the underlying biological mechanisms.

FlyTracker allows for automated analysis of walking and climbing locomotor behavior, so called geotaxis, by collecting large sequences of data over a desired time period in an entirely unbiased manner. An additional mechanical feature added to the holder rack enables movements of fly tubes to induce the startle-induced negative geotactic response. Other approaches based on digital imaging have been described previously but only with the main measure extracted from the assay being height of fly negative geotaxis [58]. A somewhat similar accuracy of walking descriptors as with FlyTracker was described in an automatic system, but it only tracked a small number of flies and just in an open horizontal field [1, 59]. Moreover, there have been some reports of automated systems which track planar fly interactions that may be useful for studies of genetic origins of fly behavior [60, 61]. In summary, the work presented demonstrates that the commercially available FlyTracker system is an excellent new tool to study fly mobility parameters with high precision (i.e., in disease models such as the PD flies here), requiring low amounts of test compounds.

## Supporting information

S1 Fig (A) The equipment and experimental setup of FlyTracker. 1. Fly tubes; 2. Fly tube holder rack with a movable frame; 3. Base frame; 4. VGA camera with the USB interface fixed at 105 mm from the center of fly tubes; 5. PC. (B) Structure of ring-fused 2-pyridones used in this work and *in vitro* thioflavin T (ThT) aggregation assay for 70 μM aS alone (filled circles) and with 100 μM FN075 (filled squares) or C10 (open circles) in 10 mM phosphate, pH 7.4 with 140 mM NaCl and 2.7 mM KCl. Experiments were performed at 37°C with continuous agitation using a 2 mm glass bead in each well. All samples contained 20 μM ThT and fluorescence was measured at 480 nm (excitation at 440 nm) in a FLUOstar Omega plate reader. (C) Densitometric analysis of Western blots (n = 3) of fly head protein extracts probed with antibody specific to human aS. The protein extracts are divided in soluble and insoluble fractions prepared as described in supplementary M&M. The aS-specific signal was normalized to its tubulin signal and then insoluble aS level was further normalized as ratio to aS soluble level. The diagram shows aS expression fold change in soluble and insoluble aS fractions. Bars represent mean values ± SD. Raw densitometric data presented in the table were acquired with Gel-Doc XR+ Imager and analysed with Image Lab 5.2 software (Bio-Rad, Richmond, CA, USA).
(TIF)

S2 Fig Compound toxicity assays. (A) Mean fraction of eclosed pupae of control (CTRL) and aS fly lines (AS) that were fed during larval stages with either vehicle (VEH, white bars) or tested compounds: FN075 (magenta bars), MS400 (green bars) or C10 (blue bars) at 100μM concentration. (B) Survival analysis was analyzed by Kaplan-Meier curves. Cumulative survival of non-expressing UAS-aS flies treated with either vehicle (CTRL TG VEH, white bars) or compounds: FN075 (CTRL TG FN075, magenta bars), MS400 (CTRL TG MS400, greean bars) and C10 (CTRL TG C10, blue bars) at 100μM concentration. (C) Mean, (D) median and (E) max lifetime for control non-expressing UAS-aS flies (CTRL TG) fed with either vehicle (VEH) or tested compounds: FN075, MS400 and C10. Numbers in bars represents mean, median and max lifetime (days). Error bars indicate ± SE.
(TIF)

S3 Fig FlyTracker acquired kinetic parameters for aS flies measured at 4, 8,15,22 and 29 days of adult drug feeding regime. Controls: vehicle treated nsyb-Gal4 outcrossed with Oregon R (CTRL VEH, dark grey bars) or aS expressing flies fed vehicle (AS VEH, white bars) or tested compounds: FN075 (AS FN075, magenta bars) or MS400 (AS MS400, green bars) or C10 (AS C10, blue bars) at 100μM concentration. Error bars indicate ± SE. *P* values are <0,05 (*), <0,01 (**), <0,001 (***). (A) Mean velocity (mm/s). (B) Maximum mean velocity (mm/s). (C) Total walking duration (s). (D) Total trajectory length (mm). (E) Fly motion (%). (F) Mean trajectory length (mm). (G) Mean number of fly walking trajectories. (H) Mean trajectory length per episode (mm).
(TIF)

S4 Fig FlyTracker acquired kinetic parameters for aS flies measured at 1, 7, 16, 21, 30 and 42 days of larval drug feeding regime. Controls: nsyb-Gal4 outcrossed with Oregon R (CTRL VEH, dark grey bars); non-expressing UAS-aS flies (CTRL TG, light grey bars). Tested compounds were either FN075 (AS FN075, magenta bars) or MS400 (AS MS400, green bars) or C10 (AS C10, blue bars) at 100μM concentration. (A) Mean velocity (mm/s). (B) Maximum velocity (mm/s). (C) Total walking duration (s). (D) Total trajectory length (mm). (E) Fly motion (%). (F) Mean trajectory length (mm). (G) Number of fly walking trajectories. (H) Mean trajectory length per episode (mm). Bars represent mean values. Error bars indicate ± SE. * *P* < 0,05; ** *P* < 0,01; *** *P* <0,001.
(TIF)

S5 Fig FlyTracker acquired kinetic parameters for non-expressing UAS-aS flies (CTRL TG) measured at 1, 7, 16, 21, and 30 days of larval drug feeding regime. Tested compounds were either FN075 (AS FN075, magenta bars) or MS400 (AS MS400, green bars) or C10 (AS C10, blue bars) at 100μM concentration and vehicle (VEH, grey bars). (A) Mean velocity (mm/s). (B) Maximum velocity (mm/s). (C) Total walking duration (s). (D) Total trajectory length (mm). (E) Fly motion (%). (F) Mean trajectory length (mm). (G) Mean number of fly walking trajectories. (H) Mean trajectory length per episode (mm). Bars represent mean values. Error bars indicate ± SE. * *P* < 0,05; ** *P* < 0,01; *** *P* <0,001.
(TIF)

S1 Table Kinetic parameters for aS and control flies. General Linear Model multivariate analysis with Fisher’s post hoc test. Significant number are highlighted in red.
(PDF)

S2 Table Pairwise comparisons and log-rank analysis of survival curves for control aS non-expressing UAS-aS flies (CTRL TG) treated either vehicle or compounds FN075, MS400, C10. Significant numbers are highlighted in red.
(PDF)

S3 Table Pairwise comparisons and log-rank analysis of survival curves for aS expressing vehicle, FN075, MS400, or C10 treated flies. Significant number are highlighted in red.
(PDF)

S4 Table Kinetic parameters for aS flies fed vehicle or compounds FN075, MS400, C10 in adult stages of life span. General Linear Model multivariate analysis with Fisher’s post hoc test. Significant numbers are highlighted in red.
(PDF)

S5 Table Kinetic parameters for aS flies fed vehicle or compounds FN075, MS400, C10 from larval stages and throughout entire life span. General Linear Model multivariate analysis with Fisher’s post hoc test. Significant numbers are highlighted in red.
(PDF)

S6 Table Kinetic parameters for control (aS non-expressing) flies fed vehicle or compounds FN075, MS400, C10 from larval stages and throughout entire life span. General Linear Model multivariate analysis with Fisher’s post hoc test. Significant numbers are highlighted in red.
(PDF)

S7 Table Effects of 2-pyridones on level of soluble aS in fly brain extracts of 20 days old flies. Multiple comparisons are presented for aS flies fed compounds FN075, MS400, C10 vs. aS flies fed vehicle (AS VEH) or vs. control aS non-expressing flies (CTRL VEH) General Linear Model multivariate analysis with Fisher’s post hoc test (upper table). Significant numbers are highlighted in red. Raw data i.e. descriptive statistics from ELISA tests are in the lower table.
(PDF)

S8 Table Kinetic parameters for aS flies fed either vehicle or L-DOPA (1mM) in adult stages. General Linear Model multivariate analysis with Fisher’s post hoc test. Significant numbers are highlighted in red.
(PDF)

S9 Table Pairwise comparisons and log-rank analysis of survival curves for aS expressing flies treated either vehicle or compounds FN075, MS400, C10 and aS non-expressing flies (CTRL VEH) vs. aS flies fed L-DOPA. Significant numbers are highlighted in red.
(PDF)

S1 Supporting Materials and Methods (PDF)
