# Supplementary figures and images for: Effects of small-molecule amyloid modulators on a Drosophila model of Parkinson’s disease
Source: PLoS One. 2017 Sep 1;12(9):e0184117. doi: 10.1371/journal.pone.0184117 (PMC5581160; doi:10.1371/journal.pone.0184117)

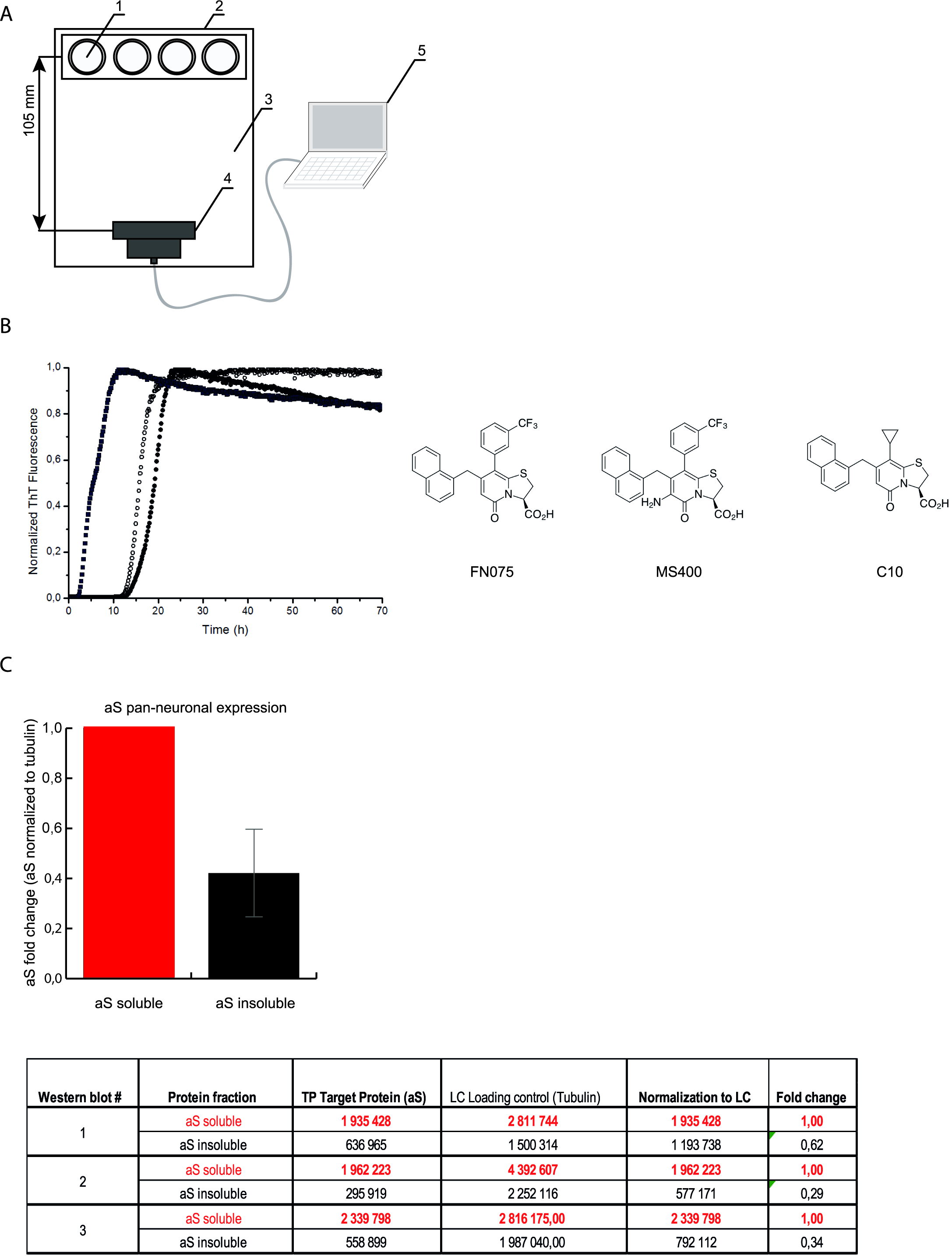

Supplement: S1 Fig — (A) The equipment and experimental setup of FlyTracker. 1. Fly tubes; 2. Fly tube holder rack with a movable frame; 3. Base frame; 4. VGA camera with the USB interface fixed at 105 mm from the center of fly tubes; 5. PC. (B) Structure of ring-fused 2-pyridones used in this work and in vitro thioflavin T (ThT) aggregation assay for 70 μM aS alone (filled circles) and with 100 μM FN075 (filled squares) or C10 (open circles) in 10 mM phosphate, pH 7.4 with 140 mM NaCl and 2.7 mM KCl. Experiments were performed at 37°C with continuous agitation using a 2 mm glass bead in each well. All samples contained 20 μM ThT and fluorescence was measured at 480 nm (excitation at 440 nm) in a FLUOstar Omega plate reader. (C) Densitometric analysis of Western blots (n = 3) of fly head protein extracts probed with antibody specific to human aS. The protein extracts are divided in soluble and insoluble fractions prepared as described in supplementary M&M. The aS-specific signal was normalized to its tubulin signal and then insoluble aS level was further normalized as ratio to aS soluble level. The diagram shows aS expression fold change in soluble and insoluble aS fractions. Bars represent mean values ± SD. Raw densitometric data presented in the table were acquired with Gel-Doc XR+ Imager and analysed with Image Lab 5.2 software (Bio-Rad, Richmond, CA, USA). (TIF) [file pone.0184117.s001.tif]

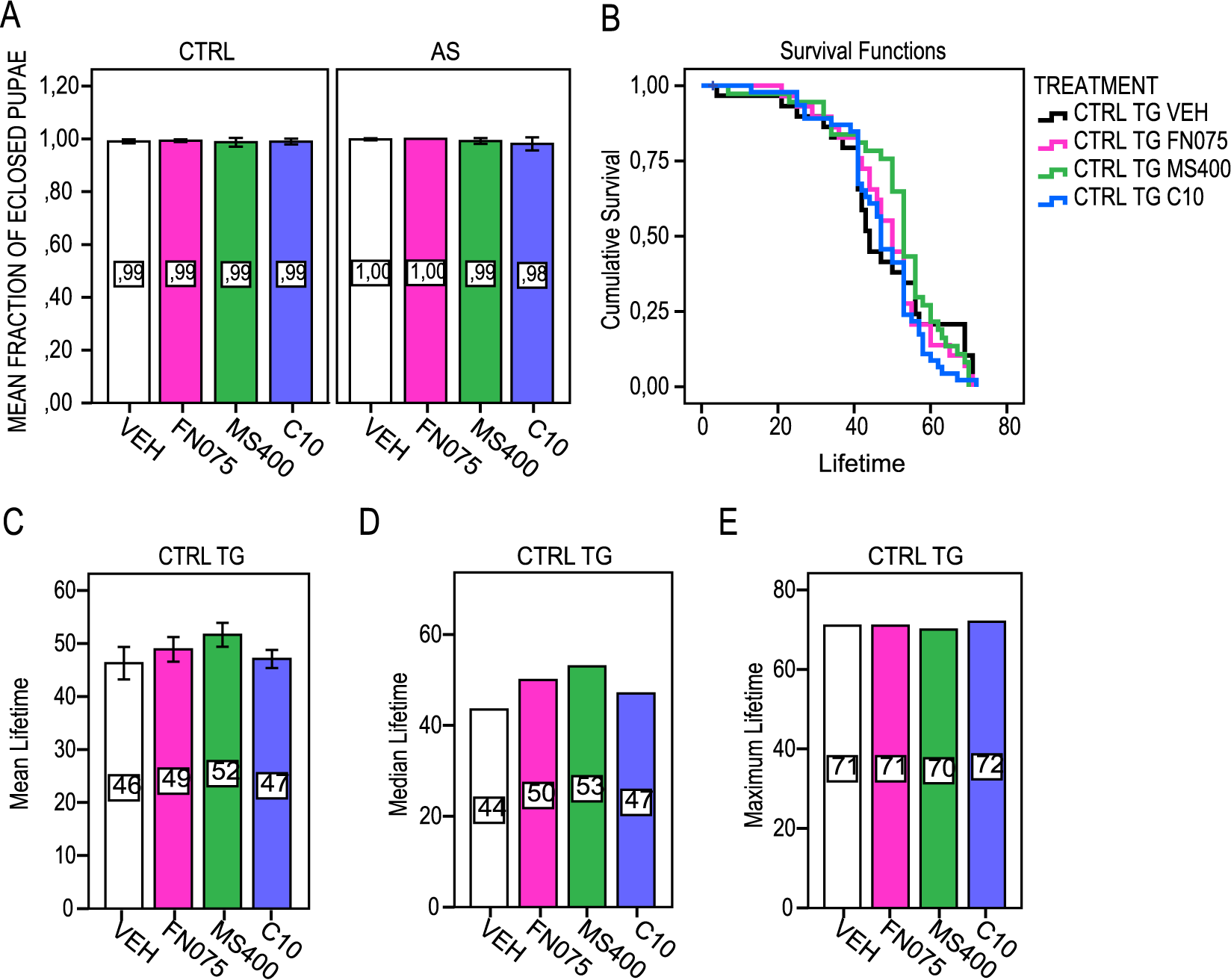

Supplement: S2 Fig — (A) Mean fraction of eclosed pupae of control (CTRL) and aS fly lines (AS) that were fed during larval stages with either vehicle (VEH, white bars) or tested compounds: FN075 (magenta bars), MS400 (green bars) or C10 (blue bars) at 100μM concentration. (B) Survival analysis was analyzed by Kaplan-Meier curves. Cumulative survival of non-expressing UAS-aS flies treated with either vehicle (CTRL TG VEH, white bars) or compounds: FN075 (CTRL TG FN075, magenta bars), MS400 (CTRL TG MS400, greean bars) and C10 (CTRL TG C10, blue bars) at 100μM concentration. (C) Mean, (D) median and (E) max lifetime for control non-expressing UAS-aS flies (CTRL TG) fed with either vehicle (VEH) or tested compounds: FN075, MS400 and C10. Numbers in bars represents mean, median and max lifetime (days). Error bars indicate ± SE. (TIF) [file pone.0184117.s002.tif]

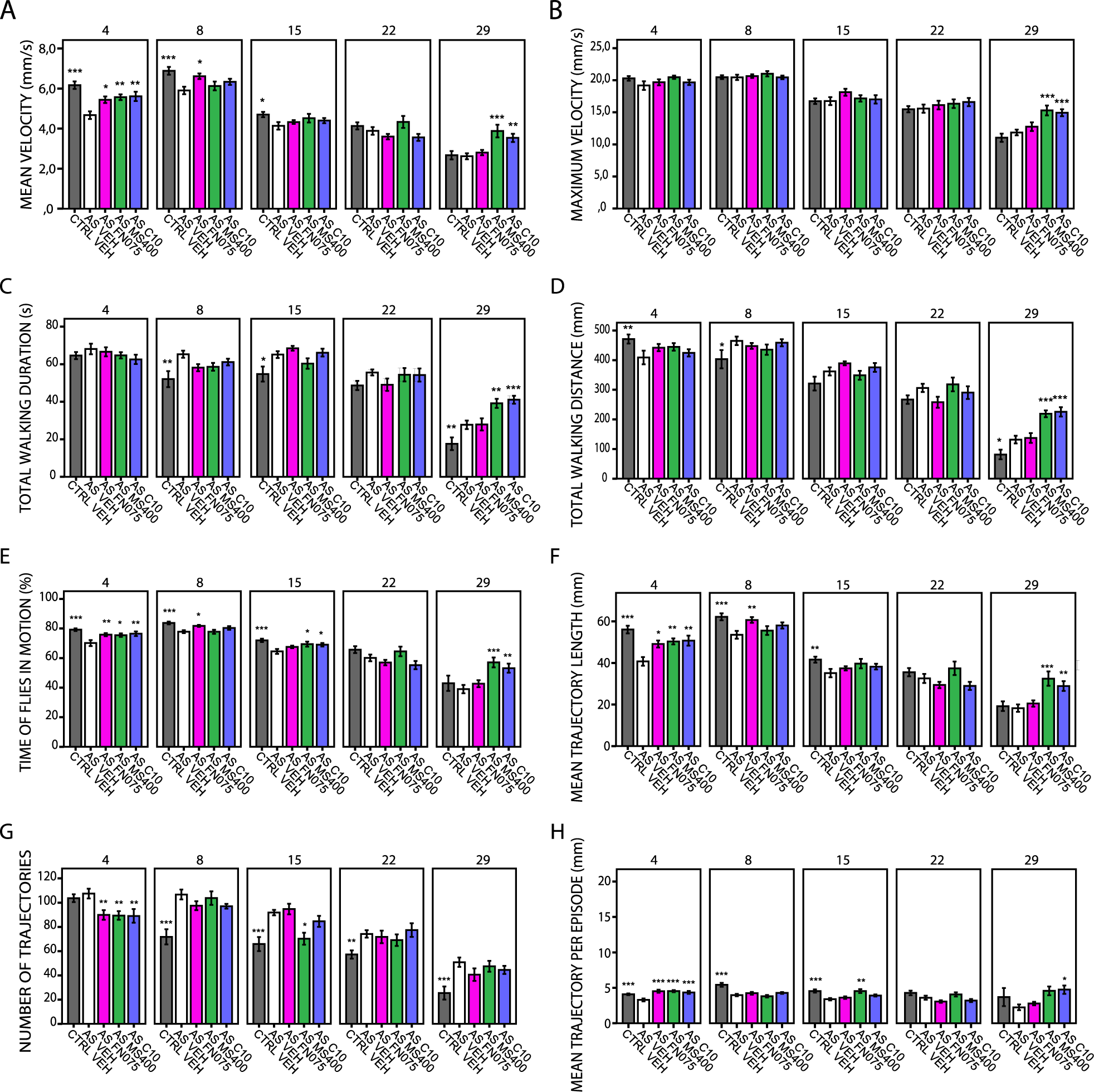

Supplement: S3 Fig — Controls: vehicle treated nsyb-Gal4 outcrossed with Oregon R (CTRL VEH, dark grey bars) or aS expressing flies fed vehicle (AS VEH, white bars) or tested compounds: FN075 (AS FN075, magenta bars) or MS400 (AS MS400, green bars) or C10 (AS C10, blue bars) at 100μM concentration. Error bars indicate ± SE. P values are <0,05 (*), <0,01 (**), <0,001 (***). (A) Mean velocity (mm/s). (B) Maximum mean velocity (mm/s). (C) Total walking duration (s). (D) Total trajectory length (mm). (E) Fly motion (%). (F) Mean trajectory length (mm). (G) Mean number of fly walking trajectories. (H) Mean trajectory length per episode (mm). (TIF) [file pone.0184117.s003.tif]

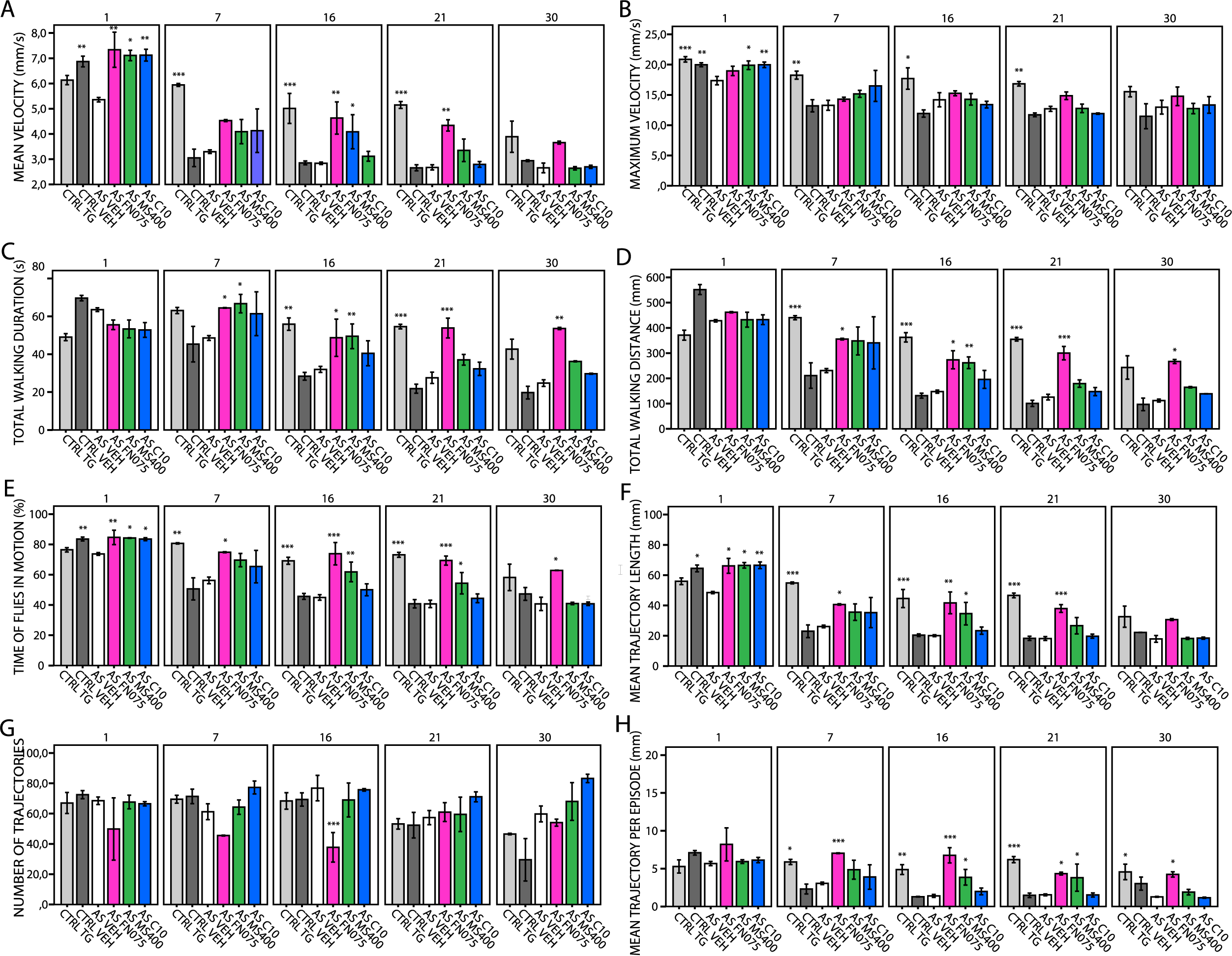

Supplement: S4 Fig — Controls: nsyb-Gal4 outcrossed with Oregon R (CTRL VEH, dark grey bars); non-expressing UAS-aS flies (CTRL TG, light grey bars). Tested compounds were either FN075 (AS FN075, magenta bars) or MS400 (AS MS400, green bars) or C10 (AS C10, blue bars) at 100μM concentration. (A) Mean velocity (mm/s). (B) Maximum velocity (mm/s). (C) Total walking duration (s). (D) Total trajectory length (mm). (E) Fly motion (%). (F) Mean trajectory length (mm). (G) Number of fly walking trajectories. (H) Mean trajectory length per episode (mm). Bars represent mean values. Error bars indicate ± SE. * P < 0,05; ** P < 0,01; *** P <0,001. (TIF) [file pone.0184117.s004.tif]

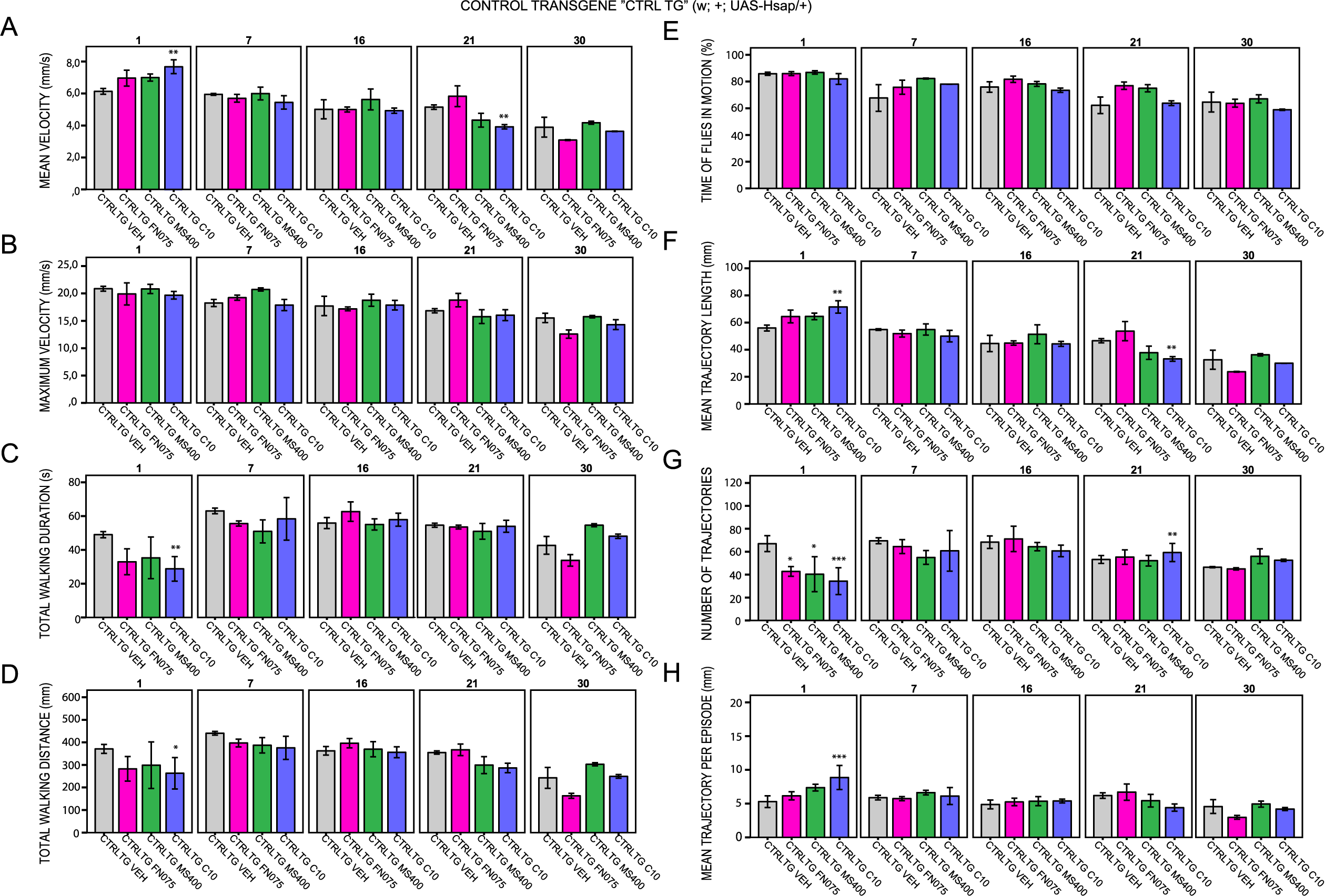

Supplement: S5 Fig — Tested compounds were either FN075 (AS FN075, magenta bars) or MS400 (AS MS400, green bars) or C10 (AS C10, blue bars) at 100μM concentration and vehicle (VEH, grey bars). (A) Mean velocity (mm/s). (B) Maximum velocity (mm/s). (C) Total walking duration (s). (D) Total trajectory length (mm). (E) Fly motion (%). (F) Mean trajectory length (mm). (G) Mean number of fly walking trajectories. (H) Mean trajectory length per episode (mm). Bars represent mean values. Error bars indicate ± SE. * P < 0,05; ** P < 0,01; *** P <0,001. (TIF) [file pone.0184117.s005.tif]
