# Supplementary material for: Effects of small-molecule amyloid modulators on a Drosophila model of Parkinson’s disease
Source: PLoS One. 2017 Sep 1;12(9):e0184117. doi: 10.1371/journal.pone.0184117 (PMC5581160; doi:10.1371/journal.pone.0184117)
Supplement: S1 Table — General Linear Model multivariate analysis with Fisher’s post hoc test. Significant number are highlighted in red. (PDF) [file pone.0184117.s006.pdf]

AS VEH	MEAN VELOCITY (mm/s)					
	1	7	16	21	30	42
CTRL VEH	0,259	0,000	0,000	0,000	0,080	0,000
**AS VEH**	**MAXIMUM VELOCITY (mm/s)**					
	1	7	16	21	30	42
CTRL VEH	0,001	0,002	0,021	0,003	0,166	0,399
**AS VEH**	**TOTAL DURATION (S)**					
	1	7	16	21	30	42
CTRL VEH	0,172	0,069	0,002	0,000	0,085	0,115
**AS VEH**	**TOTAL TRAJECTORY (mm)**					
	1	7	16	21	30	42
CTRL VEH	0,493	0,001	0,000	0,000	0,059	0,008
**AS VEH**	**MOTION (%)**					
	1	7	16	21	30	42
CTRL VEH	0,506	0,002	0,000	0,000	0,075	0,001
**AS VEH**	**MEAN TRAJECTORY LENGTH (mm)**					
	1	7	16	21	30	42
CTRL VEH	0,281	0,000	0,000	0,000	0,065	0,000
**AS VEH**	**NUMBER OF TRAJECTORIES**					
	1	7	16	21	30	42
CTRL VEH	0,917	0,291	0,403	0,679	0,305	0,000
**AS VEH**	**MEAN TRAJECTORY PER EPISODE (mm)**					
	1	7	16	21	30	42
CTRL VEH	0,867	0,012	0,002	0,000	0,042	0,000
