# Supplementary material for: Effects of small-molecule amyloid modulators on a Drosophila model of Parkinson’s disease
Source: PLoS One. 2017 Sep 1;12(9):e0184117. doi: 10.1371/journal.pone.0184117 (PMC5581160; doi:10.1371/journal.pone.0184117)
Supplement: S2 Table — Significant numbers are highlighted in red. (PDF) [file pone.0184117.s007.pdf]

Pairwise Comparisons									
		**AS TG VEH**		**AS TG FN075**		**AS TG MS400**		**AS TG C10**	
	**TREATMENT**	Chi-Square	Sig.	Chi-Square	Sig.	Chi-Square	Sig.	Chi-Square	Sig.
**Log Rank (Mantel-Cox)**	**AS TG VEH**			,059	,807	,047	,827	,441	,506
	**AS TG FN075**	,059	,807			,848	,357	,460	,498
	**AS TG MS400**	,047	,827	,848	,357			4,166	,041
	**AS TG C10**	,441	,506	,460	,498	4,166	,041		
