# Supplementary material for: Effects of small-molecule amyloid modulators on a Drosophila model of Parkinson’s disease
Source: PLoS One. 2017 Sep 1;12(9):e0184117. doi: 10.1371/journal.pone.0184117 (PMC5581160; doi:10.1371/journal.pone.0184117)
Supplement: S3 Table — Significant number are highlighted in red. (PDF) [file pone.0184117.s008.pdf]

Pairwise Comparisons											
**TREATMENT**		**AS VEH**		**CTRL VEH**		**AS FN075**		**AS MS400**		**AS C10**	
		Chi-Square	Sig.	Chi-Square	Sig.	Chi-Square	Sig.	Chi-Square	Sig.	Chi-Square	Sig.
Log Rank (Mantel-Cox)	**AS VEH**			7,002	,008	13,703	,000	18,617	,000	10,573	,001
	**CTRL VEH**	7,002	,008			3,964	,046	34,526	,000	34,062	,000
	**AS FN075**	13,703	,000	3.964	,046			35,673	,000	36,893	,000
	**AS MS400**	18,617	,000	34,526	,000	35,673	,000			4,648	,031
	**AS C10**	10,573	,001	34,062	,000	36,893	,000	4,648	,031		
