# Supplementary material for: Effects of small-molecule amyloid modulators on a Drosophila model of Parkinson’s disease
Source: PLoS One. 2017 Sep 1;12(9):e0184117. doi: 10.1371/journal.pone.0184117 (PMC5581160; doi:10.1371/journal.pone.0184117)
Supplement: S4 Table — General Linear Model multivariate analysis with Fisher’s post hoc test. Significant numbers are highlighted in red. (PDF) [file pone.0184117.s009.pdf]

AS VEH	MEAN VELOCITY (mm/s)				
	4	8	15	22	29
AS FN075	0,024	0,013	0,461	0,342	0,544
AS MS400	0,009	0,421	0,146	0,134	0,000
AS C10	0,006	0,125	0,321	0,280	0,004
CTRL VEH	0,000	0,000	0,012	0,362	0,874
**AS VEH**	**MAXIMUM VELOCITY (mm/s)**				
	4	8	15	22	29
AS FN075	0,488	0,691	0,120	0,536	0,297
AS MS400	0,077	0,263	0,639	0,391	0,000
AS C10	0,494	0,990	0,772	0,253	0,001
CTRL VEH	0,070	0,933	0,975	0,907	0,350
**AS VEH**	**TOTAL DURATION (s)**				
	4	8	15	22	29
AS FN075	0,691	0,137	0,566	0,124	0,955
AS MS400	0,377	0,163	0,418	0,778	0,004
AS C10	0,149	0,385	0,861	0,745	0,001
CTRL VEH	0,275	0,003	0,039	0,080	0,013
**AS VEH**	**TOTAL TRAJECTORY (mm)**				
	4	8	15	22	29
AS FN075	0,233	0,612	0,426	0,072	0,805
AS MS400	0,199	0,384	0,711	0,652	0,000
AS C10	0,575	0,856	0,693	0,550	0,000
CTRL VEH	0,009	0,048	0,164	0,111	0,020
**AS VEH**	**MOTION (%)**				
	4	8	15	22	29
AS FN075	0,007	0,015	0,171	0,398	0,429
AS MS400	0,011	0,961	0,029	0,234	0,000
AS C10	0,003	0,112	0,044	0,193	0,004
CTRL VEH	0,000	0,000	0,000	0,107	0,407
**AS VEH**	**MEAN TRAJECTORY LENGTH (mm)**				
	4	8	15	22	29
AS FN075	0,015	0,008	0,411	0,340	0,505
AS MS400	0,006	0,432	0,105	0,144	0,000
AS C10	0,004	0,087	0,267	0,276	0,003
CTRL VEH	0,000	0,000	0,007	0,324	0,783
**AS VEH**	**NUMBER OF TRAJECTORIES**				
	4	8	15	22	29
AS FN075	0,009	0,225	0,753	0,694	0,126
AS MS400	0,007	0,692	0,019	0,419	0,610
AS C10	0,006	0,201	0,424	0,615	0,338
CTRL VEH	0,490	0,000	0,001	0,005	0,000
**AS VEH**	**MEAN TRAJECTORY PER EPISODE (mm)**				
	4	8	15	22	29
AS FN075	0,000	0,436	0,562	0,199	0,668
AS MS400	0,000	0,654	0,004	0,273	0,069
AS C10	0,000	0,372	0,167	0,339	0,050
CTRL VEH	0,001	0,000	0,001	0,089	0,263
