# Supplementary material for: Effects of small-molecule amyloid modulators on a Drosophila model of Parkinson’s disease
Source: PLoS One. 2017 Sep 1;12(9):e0184117. doi: 10.1371/journal.pone.0184117 (PMC5581160; doi:10.1371/journal.pone.0184117)
Supplement: S5 Table — General Linear Model multivariate analysis with Fisher’s post hoc test. Significant numbers are highlighted in red. (PDF) [file pone.0184117.s010.pdf]

AS VEH	MEAN VELOCITY (mm/s)					
	1	7	16	21	30	42
AS FN075	0,005	0,056	0,008	0,002	0,118	0,833
AS MS400	0,013	0,251	0,020	0,184	0,982	0,104
AS C10	0,008	0,229	0,600	0,815	0,952	n/a
CTRL VEH	0,016	0,692	0,977	0,975	0,678	n/a
**AS VEH**	**MAXIMUM VELOCITY (mm/s)**					
	1	7	16	21	30	42
AS FN075	0,134	0,488	0,523	0,130	0,283	0,012
AS MS400	0,019	0,240	0,960	0,958	0,900	0,861
AS C10	0,011	0,052	0,555	0,557	0,838	n/a
CTRL VEH	0,007	0,953	0,084	0,451	0,412	n/a
**AS VEH**	**TOTAL DURATION (s)**					
	1	7	16	21	30	42
AS FN075	0,451	0,047	0,045	0,000	0,004	0,958
AS MS400	0,336	0,039	0,009	0,154	0,262	0,054
AS C10	0,287	0,138	0,197	0,473	0,624	n/a
CTRL VEH	0,516	0,671	0,568	0,371	0,619	n/a
**AS VEH**	**TOTAL TRAJECTORY (mm)**					
	1	7	16	21	30	42
AS FN075	0,684	0,039	0,016	0,000	0,017	0,726
AS MS400	0,961	0,074	0,007	0,241	0,434	0,023
AS C10	0,958	0,093	0,240	0,630	0,690	n/a
CTRL VEH	0,101	0,735	0,685	0,574	0,818	n/a
**AS VEH**	**MOTION (%)**					
	1	7	16	21	30	42
AS FN075	0,009	0,015	0,000	0,000	0,017	0,885
AS MS400	0,012	0,099	0,002	0,042	0,978	0,236
AS C10	0,014	0,255	0,347	0,579	0,994	n/a
CTRL VEH	0,009	0,440	0,894	0,987	0,493	n/a
**AS VEH**	**MEAN TRAJECTORY LENGTH (mm)**					
	1	7	16	21	30	42
AS FN075	0,013	0,044	0,004	0,001	0,078	0,854
AS MS400	0,011	0,221	0,014	0,142	0,970	0,095
AS C10	0,008	0,234	0,565	0,790	0,951	n/a
CTRL VEH	0,012	0,649	0,949	0,985	0,578	n/a
**AS VEH**	**NUMBER OF TRAJECTORIES**					
	1	7	16	21	30	42
AS FN075	0,198	0,050	0,001	0,725	0,623	0,000
AS MS400	0,954	0,724	0,389	0,828	0,520	0,035
AS C10	0,883	0,067	0,901	0,170	0,075	n/a
CTRL VEH	0,756	0,199	0,394	0,605	0,025	n/a
**AS VEH**	**MEAN TRAJECTORY PER EPISODE (mm)**					
	1	7	16	21	30	42
AS FN075	0,279	0,001	0,000	0,021	0,045	0,736
AS MS400	0,912	0,127	0,013	0,049	0,687	0,047
AS C10	0,837	0,468	0,542	0,983	0,944	n/a
CTRL VEH	0,495	0,479	0,911	0,954	0,263	n/a
