# Supplementary material for: Effects of small-molecule amyloid modulators on a Drosophila model of Parkinson’s disease
Source: PLoS One. 2017 Sep 1;12(9):e0184117. doi: 10.1371/journal.pone.0184117 (PMC5581160; doi:10.1371/journal.pone.0184117)
Supplement: S6 Table — General Linear Model multivariate analysis with Fisher’s post hoc test. Significant numbers are highlighted in red. (PDF) [file pone.0184117.s011.pdf]

CTRL TG VEH	MEAN VELOCITY (mm/s)					
	1	7	16	21	30	42
CTRL TG FN075	0,185	0,661	0,981	0,115	0,239	n.d.
CTRL TG MS400	0,123	0,934	0,237	0,075	0,681	n.d.
CTRL TG C10	0,002	0,421	0,860	0,005	0,707	n.d.
**CTRL TG VEH**	**MAXIMUM VELOCITY (mm/s)**					
	**1**	**7**	**16**	**21**	**30**	**42**
CTRL TG FN075	0,528	0,476	0,701	0,066	0,077	n.d.
CTRL TG MS400	0,971	0,105	0,409	0,340	0,891	n.d.
CTRL TG C10	0,328	0,803	0,901	0,449	0,460	n.d.
**CTRL TG VEH**	**TOTAL DURATION (s)**					
	**1**	**7**	**16**	**21**	**30**	**42**
CTRL TG FN075	0,071	0,345	0,396	0,859	0,363	n.d.
CTRL TG MS400	0,084	0,173	0,909	0,581	0,220	n.d.
CTRL TG C10	0,005	0,595	0,781	0,921	0,578	n.d.
**CTRL TG VEH**	**TOTAL TRAJECTORY (mm)**					
	**1**	**7**	**16**	**21**	**30**	**42**
CTRL TG FN075	0,165	0,447	0,553	0,780	0,252	n.d.
CTRL TG MS400	0,204	0,403	0,896	0,235	0,390	n.d.
CTRL TG C10	0,035	0,310	0,899	0,122	0,926	n.d.
**CTRL TG VEH**	**MOTION (%)**					
	**1**	**7**	**16**	**21**	**30**	**42**
CTRL TG FN075	0,969	0,322	0,636	0,308	0,284	n.d.
CTRL TG MS400	0,764	0,295	0,192	0,118	0,278	n.d.
CTRL TG C10	0,318	0,702	0,437	0,053	0,929	n.d.
**CTRL TG VEH**	**MEAN TRAJECTORY LENGTH (mm)**					
	**1**	**7**	**16**	**21**	**30**	**42**
CTRL TG FN075	0,214	0,627	0,962	0,138	0,238	n.d.
CTRL TG MS400	0,164	0,996	0,238	0,078	0,627	n.d.
CTRL TG C10	0,005	0,474	0,951	0,005	0,728	n.d.
**CTRL TG VEH**	**NUMBER OF TRAJECTORIES**					
	**1**	**7**	**16**	**21**	**30**	**42**
CTRL TG FN075	0,045	0,642	0,792	0,138	0,909	n.d.
CTRL TG MS400	0,014	0,229	0,694	0,078	0,471	n.d.
CTRL TG C10	0,001	0,467	0,428	0,465	0,649	n.d.
**CTRL TG VEH**	**MEAN TRAJECTORY LENGTH PER EPISODE (mm)**					
	**1**	**7**	**16**	**21**	**30**	**42**
CTRL TG FN075	0,527	0,906	0,763	0,595	0,285	n.d.
CTRL TG MS400	0,090	0,587	0,673	0,448	0,808	n.d.
CTRL TG C10	0,001	0,860	0,643	0,059	0,802	n.d.
