# Supplementary material for: Effects of small-molecule amyloid modulators on a Drosophila model of Parkinson’s disease
Source: PLoS One. 2017 Sep 1;12(9):e0184117. doi: 10.1371/journal.pone.0184117 (PMC5581160; doi:10.1371/journal.pone.0184117)
Supplement: S7 Table — Multiple comparisons are presented for aS flies fed compounds FN075, MS400, C10 vs. aS flies fed vehicle (AS VEH) or vs. control aS non-expressing flies (CTRL VEH) General Linear Model multivariate analysis with Fisher’s post hoc test (upper table). Significant numbers are highlighted in red. Raw data i.e. descriptive statistics from ELISA tests are in the lower table. (PDF) [file pone.0184117.s012.pdf]

Multiple comparisons						
Dependent Variable: soluble aS (ng aS/mg protein)						
(I) TREATMENT	(J) TREATEMNT	Mean Difference (I-J)	Std. Error	Sig.	95% Confidence Interval	
					Lower Bound	Upper Bound
AS VEH	AS FN075	28,3485	13,19089	0,084	-5,5597	62,2568
	AS MS400	-125,7983*	13,19089	0,000	-159,7066	-91,89
	AS C10	-80,0952*	13,19089	0,002	-114,0034	-46,1869
	CTRL VEH	123,3651*	13,19089	0,000	89,4568	157,2733
CTRL VEH	AS VEH	-123,3651*	13,19089	0,000	-157,2733	-89,4568
	AS FN075	-95,0165*	13,19089	0,001	-128,9248	-61,1083
	AS MS400	-249,1633*	13,19089	0,000	-283,0716	-215,2551
	AS C10	-203,4602*	13,19089	0,000	-237,3685	-169,552

Descriptive Statistics			
Dependent Variable: soluble aS (ng aS/mg protein)			
**TREATMENT**	**Mean**	**Std. Deviation**	**N**
CTRL VEH	0,00	0	2
AS VEH	123,37	2,74	2
AS FN075	95,02	7,65	2
AS MS400	249,16	7,00	2
AS C10	203,46	27,48	2
