# Supplementary material for: Effects of small-molecule amyloid modulators on a Drosophila model of Parkinson’s disease
Source: PLoS One. 2017 Sep 1;12(9):e0184117. doi: 10.1371/journal.pone.0184117 (PMC5581160; doi:10.1371/journal.pone.0184117)
Supplement: S8 Table — General Linear Model multivariate analysis with Fisher’s post hoc test. Significant numbers are highlighted in red. (PDF) [file pone.0184117.s013.pdf]

AS VEH	MEAN VELOCITY (mm/s)				
	4	8	15	22	29
AS DOPA	0,008	0,114	0,004	0,410	0,000
**AS VEH**	**MAXIMUM VELOCITY (mm/s)**				
	4	8	15	22	29
AS DOPA	0,138	0,892	0,071	0,300	0,010
**AS VEH**	**TOTAL DURATION (S)**				
	4	8	15	22	29
AS DOPA	0,263	0,317	0,228	0,071	0,634
**AS VEH**	**TOTAL TRAJECTORY (mm)**				
	4	8	15	22	29
AS DOPA	0,466	0,578	0,951	0,234	0,509
**AS VEH**	**MOTION (%)**				
	4	8	15	22	29
AS DOPA	0,002	0,022	0,011	0,478	0,000
**AS VEH**	**MEAN TRAJECTORY LENGTH (mm)**				
	4	8	15	22	29
AS DOPA	0,005	0,081	0,004	0,409	0,000
**AS VEH**	**NUMBER OF TRAJECTORIES**				
	4	8	15	22	29
AS DOPA	0,003	0,280	0,195	0,139	0,008
**AS VEH**	**MEAN TRAJECTORY PER EPISODE (mm)**				
	4	8	15	22	29
AS DOPA	0,000	0,511	0,068	0,565	0,000
