# Supplementary material for: Effects of small-molecule amyloid modulators on a Drosophila model of Parkinson’s disease
Source: PLoS One. 2017 Sep 1;12(9):e0184117. doi: 10.1371/journal.pone.0184117 (PMC5581160; doi:10.1371/journal.pone.0184117)
Supplement: S9 Table — Significant numbers are highlighted in red. (PDF) [file pone.0184117.s014.pdf]

Pairwise Comparisons			
**TREATMENT**		**AS DOPA**	
		Chi-Square	Sig.
Log Rank (Mantel-Cox)	**AS VEH**	7,714	,005
	**AS FN075**	0,303	,582
	**AS MS400**	32,628	,000
	**AS C10**	27,538	,000
	**CTRL VEH**	0,738	,390
