# Supplementary material for: Effects of small-molecule amyloid modulators on a Drosophila model of Parkinson’s disease
Source: PLoS One. 2017 Sep 1;12(9):e0184117. doi: 10.1371/journal.pone.0184117 (PMC5581160; doi:10.1371/journal.pone.0184117)
Supplement: S1 Supporting Materials and Methods — (PDF) [file pone.0184117.s015.pdf]

Supporting Material and Methods.

Protein extraction and quantification of α-synuclein levels

For immunoblot technique, protein extracts were prepared according to protocol modified from [1]. Twenty fly heads of 20 days old flies were homogenized in 70 µl of extraction buffer (20 mM Tris pH 7.6, 50 mM NaCl, 1% Triton X-100, Protease inhibitor (Amresco LLC, OH)), vortexed gently and incubated on ice for 30 min. After centrifugation for 60 min at 15 x 1000 g in +4°C, supernatants were collected and mixed with 4 x LDS Sample Buffer and DTT containing (10 x) Sample Reducing Agent (Life Technologies, Carlsbad, CA). The remaining pellets were resuspended in SDS extraction buffer (50 mM Tris pH 7.6, 5 mM EDTA, 4% SDS), vortexed and boiled for 10 min. Supernatants were collected after centrifugation 10 min at 15 x 1000 g and mixed with 4 x LDS sample buffer + DTT as described above. Both fractions were boiled for 20 min before electrophoresis.
For each extract a volume corresponding to 1,5 fly head was resolved on NuPAGE® Novex® 4-12% Bis-Tris Protein Gel in MES SDS running buffer and electroblotted onto Nitrocellulose membrane using iBlot2 gel transfer device (Life Technologies). All steps were performed according to the manufacturer. The primary antibodies used were mouse monoclonal against α-tubulin (1:5000, clone B-5-1-2, Life technologies) and rabbit polyclonal against human aS 1:1000 (AlexoTech AB). Detection was performed with Western Breeze Chromogenic kit anti-mouse or anti-rabbit, respectively. aS levels were quantified with Gel-Doc XR+ Imager and Image Lab 5.2 software (Bio-Rad, Richmond, CA, USA).

For ELISA, five fly heads of 10 days drug fed flies were homogenized in 50 µl extraction buffer (1X PBS pH 7.0, 5mM EDTA) with protease inhibitors coctail (Amresco LLC, OH), vortexed gently and incubated at RT for 10 min. Following centrifugation at 12 000 g for 10 min, supernatants were transferred to fresh tubes as the soluble fraction and then sonicated in ice-bath for 10 min. Remaining pellet was discarded. Detection and quantification of α-synuclein in samples were performed with α-Synuclein ELISA Kit, Human (ThermoFisher Scientific) according to the manufacturer. Briefly, samples containing α-synuclein protein extracted from fly heads were diluted 1:5 with Standard Diluent Buffer and duplicate 50 µl aliquots were added to the appropriate wells on microtitre plate. The reaction was started by adding 50 µl of Hu α-synuclein Detection Antibody solution. Following incubation for 3 hours at RT with shaking, the plate was washed four times with 1X Wash Buffer and 100 µl of 1X Anti-Rabbit IgG HRP was added into each well. The next step was incubation for 30 min and washing wells 4 times with 1X Wash Buffer afterwards. Then 100 µl of Stabilized Chromogen was added and after 30 min of incubation step in the dark 100 µl of Stop Solution was added. The measurement was taken in a Thermo Scientific Multiscan GO.

References

1. Auluck, P.K., M.C. Meulener, and N.M. Bonini, *Mechanisms of Suppression of {alpha}-Synuclein Neurotoxicity by Geldanamycin in Drosophila.* J Biol Chem, 2005. **280**(4): p. 2873-8.
